# Ferroptosis: principles and significance in health and disease

**DOI:** 10.1186/s13045-024-01564-3

**Published:** 2024-06-06

**Authors:** Fangquan Chen, Rui Kang, Daolin Tang, Jiao Liu

**Affiliations:** 1 DAMP Laboratory, The Third Affiliated Hospital of Guangzhou Medical University, Guangzhou, 510150 Guangdong China; 2 Department of Surgery, UT Southwestern Medical Center, Dallas, Texas 75390 USA

**Keywords:** Biomarker, Cancer therapy, Ferroptosis, Human disease, Immunity

## Abstract

Ferroptosis, an iron-dependent form of cell death characterized by uncontrolled lipid peroxidation, is governed by molecular networks involving diverse molecules and organelles. Since its recognition as a non-apoptotic cell death pathway in 2012, ferroptosis has emerged as a crucial mechanism in numerous physiological and pathological contexts, leading to significant therapeutic advancements across a wide range of diseases. This review summarizes the fundamental molecular mechanisms and regulatory pathways underlying ferroptosis, including both GPX4-dependent and -independent antioxidant mechanisms. Additionally, we examine the involvement of ferroptosis in various pathological conditions, including cancer, neurodegenerative diseases, sepsis, ischemia–reperfusion injury, autoimmune disorders, and metabolic disorders. Specifically, we explore the role of ferroptosis in response to chemotherapy, radiotherapy, immunotherapy, nanotherapy, and targeted therapy. Furthermore, we discuss pharmacological strategies for modulating ferroptosis and potential biomarkers for monitoring this process. Lastly, we elucidate the interplay between ferroptosis and other forms of regulated cell death. Such insights hold promise for advancing our understanding of ferroptosis in the context of human health and disease.

## Introduction

In 1842, Karl Vogt observed the metamorphosis of tadpoles and noted the disappearance of the tadpole notochord during development, marking one of the earliest recognitions of cell death in human observation [1]. Vogt’s realization that the disappearance of specific cells held physiological significance for development highlighted the phenomenon of cell death for the first time. However, technological limitations hindered the elucidation of the ultrastructural characteristics of cell death until 1972, thereby delaying the comprehension of programmed cell death, commonly referred to as apoptosis [2]. Programmed cell death plays a crucial role in normal tissue development, immune cell selection, and the elimination of damaged and infected cells. As various forms of stress-related cell death became recognized, cell death is currently broadly categorized into accidental cell death (ACD), an uncontrolled biological process triggered by irreversible external stimuli, and regulated cell death (RCD), governed by molecular network mechanisms and susceptible to modulation by experimental compounds or clinical drugs [3–5].

Cell membranes, comprising plasma and organelle membranes, regulate the integration of internal and external environmental stimuli alongside genetic signaling. These include radiation exposure, various nutrient signals, maintenance of redox homeostasis, immunosurveillance, intercellular communication, and tumor-control signaling, ultimately dictating the cell’s fate in terms of survival or demise [6, 7]. In this context, ferroptosis emphasizes the significance of membranes as it signifies a form of non-apoptotic RCD induced by uncontrolled lipid peroxidation [8]. Since the coining of the term “ferroptosis” in the discovery of agents selectively targeting RAS proto-oncogene, GTPase (*RAS*) mutation cancer cells in 2012 [9], the dysregulation of ferroptotic ecosystem has become implicated in various physiological and pathological conditions [10]. Consequently, it has emerged as a therapeutic target for numerous human diseases [11, 12].

In this review, we provide an overview of the fundamental mechanisms driving ferroptosis and the corresponding defensive mechanisms. We examine the interactions between ferroptosis and immunity, delineating their implications for various human diseases, particularly in the context of cancer therapy. Additionally, we summarize the primary pharmacological approaches and potential biomarkers associated with ferroptosis.

## The core mechanism of ferroptosis

**Fig. 1:**
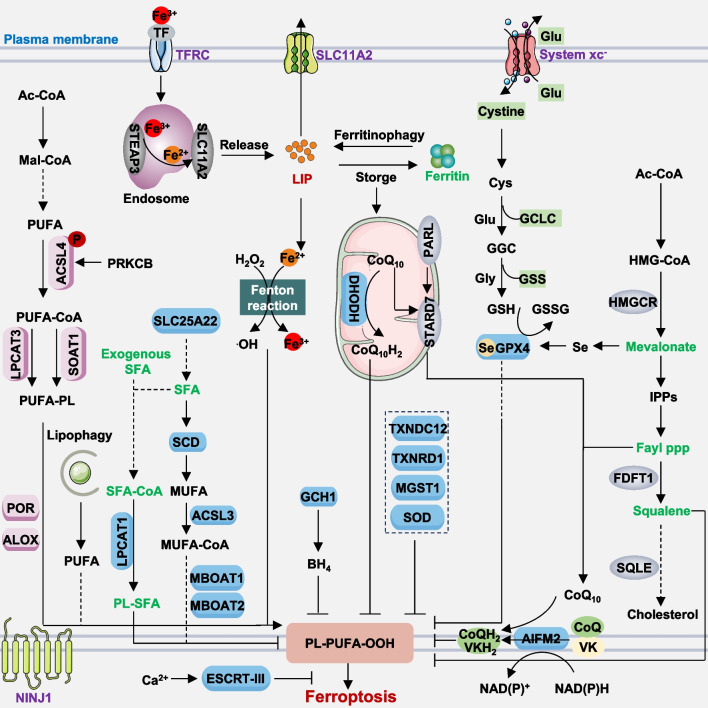
Molecular mechanisms of ferroptosis. Ferroptosis is a form of regulated cell death characterized by iron-dependent lipid peroxidation and subsequent plasma membrane rupture. It can occur via two primary pathways: the extrinsic pathway, which relies on transporters such as activating TFRC or inhibiting system xc-, and the intrinsic pathway, which is enzyme-regulated, for example, by inhibiting GPX4 or AIFM2. Ferroptosis arises from an imbalance between oxidants and antioxidants, driven by abnormal expression and activity of various redox-active enzymes that either produce or neutralize free radicals and lipid oxidation products. The plasma membrane damage can be repaired by the NINJ1 protein or ESCRT-III machinery

Elevated iron accumulation and excessive lipid peroxidation serve as central triggers of ferroptosis, with the chelation of surplus iron and the activation of both glutathione peroxidase 4 (GPX4)-dependent and -independent antioxidant pathways acting as inhibitory mechanisms against ferroptosis (Fig. 1). Hence, at the core of ferroptosis lies a disruption in redox homeostasis, as elaborated below.

### Lipid peroxidation

Lipid peroxidation is a biochemical process where free radicals, such as reactive oxygen species (ROS), attack and oxidize lipids in cell membranes and/or membrane organelles [13]. This process can lead to the formation of lipid peroxides and other reactive compounds, which can further propagate oxidative damage to nearby molecules. Increased production or stimulation of ROS can induce lipid peroxidation, resulting in various forms of RCD [14–16]. ROS are chemically reactive molecules containing oxygen, generated from various sources, including but not limited to mitochondrial respiratory chain [17], the Fenton reaction [18], nicotinamide adenine dinucleotide phosphate (NADPH) oxidase (NOX) [19], and the enzymatic reaction [20, 21]. These ROS can initiate and/or enhance ferroptosis susceptibility across different cell types or tissues [22].

#### Mitochondria ROS

Mitochondria generates ROS as inherent byproducts of the electron transport chain (ETC) during cellular respiration. The ETC comprises a sequence of protein complexes situated within the inner mitochondrial membrane. In the process of cellular respiration, electrons are sequentially transferred along the ETC, ultimately reaching oxygen molecules. Electrons escape prematurely from the ETC, particularly from complexes I and III, due to factors, such as imperfect electron flow coupling or mitochondrial membrane damage. These leaked electrons can engage with molecular oxygen (O_2_) within the mitochondria, resulting in the formation of partially reduced oxygen species known as superoxide radicals (O_2_^·−^). Superoxide dismutase enzymes can then convert superoxide into hydrogen peroxide (H_2_O_2_). The original study suggests that mitochondrial ROS may not be crucial for inducing ferroptosis because cells depleted of mitochondrial DNA, known as ρ0 cells, which lack critical respiratory chain catalytic subunits, still exhibit sensitivity to ferroptosis activators [9]. Recent studies suggest that phospholipids containing diacyl-polyunsaturated fatty acid (PC-PUFA_2_s) tails may enhance ferroptosis sensitivity in cancer cells by directly interacting with the mitochondrial ETC, thereby promoting the generation of ROS to initiate lipid peroxidation [23]. H_2_O_2_ was previously recognized as a broadly effective apoptosis inducer, but recent studies also show its ability to induce ferroptosis [24]. However, the precise downstream pathways by which mitochondrial ROS production (e.g., H_2_O_2_) contribute to the initiation of apoptosis or ferroptosis remain inadequately elucidated. One possible hypothesis is that the release of various mitochondrial proteins may drive distinct cell death pathways [25]. It is also crucial to identify the ranges within which these pathways are primarily activated.

#### Fenton reaction

The Fenton reaction is a chemical reaction that involves the catalytic conversion of H_2_O_2_ into hydroxyl radicals (^·^OH) in the presence of transition metal ions, typically iron (II). The hydroxyl radicals are highly reactive and can initiate various oxidative processes, including the peroxidation of lipids, DNA damage, and protein oxidation. In the context of lipid peroxidation, labile iron catalyzes the formation of lipid hydroxyl radicals (LO^·^) and lipid peroxyl radicals (LOO^·^) from phospholipid hydroperoxides (LOOH) [26]. Hence, the modulation of iron homeostasis can impact the susceptibility to ferroptosis, with this influence contingent upon the regulation of iron uptake, utilization, storage, and export within cells and tissues [27]. Moreover, arginine undergoes conversion into polyamines, exerting ferroptosis-promoting effects in an H_2_O_2_-dependent manner [28]. Iron overload signaling triggers the upregulation of ornithine decarboxylase 1 (ODC1), a critical enzyme facilitating polyamine synthesis, through the WNT-MYC proto-oncogene, BHLH transcription factor (MYC) pathway [28]. This leads to heightened polyamine production, establishing a positive feedback loop of iron overload-WNT-MYC-ODC1-polyamine-H_2_O_2_, thereby amplifying the ferroptosis response across various cancer cell lines including HT-1080, A549, H1299, PC9, H23, and in vivo contexts [28].

#### NADPH oxidase (NOX)

The NOX family comprises enzymes that catalyze the generation of O_2_^·−^ by transferring electrons from NADPH to molecular oxygen. It encompasses several members, including NOX1, NOX2, NOX3, NOX4, NOX5, and the dual oxidase 1 (DUOX1) and DUOX2, each with distinct tissue distributions, cellular localization, and regulatory mechanisms. NOX enzymes play pivotal roles in the immune response, where they contribute to gut microbiota elimination by producing ROS [29]. Additionally, they participate in various signaling pathways, including those associated with ferroptosis [30, 31]. For instance, recent studies have suggested that 4-hydroxynonenal (4HNE), a product of lipid peroxidation, can activate NOX1, thereby establishing a positive feedback loop that amplifies ferroptosis sensitivity in cancer cells (e.g., HT-1080 and PANC-1) [19]. Further investigation is warranted to elucidate the specific contributions of NOX-mediated ROS production to ferroptotic cell death and to explore the potential of targeting these enzymes for therapeutic interventions in diseases where ferroptosis is implicated.

#### Enzymatic reactions

The enzyme-dependent pathway represents one of the most extensive and pivotal mechanisms driving lipid peroxidation. It involves multiple key enzymes, notably acyl-coenzyme A (CoA) synthetase long chain family member 4 (ACSL4) and lysophosphatidylcholine acyltransferase 3 (LPCAT3) [32–35]. ACSL4 orchestrates two distinct pathways to catalyze the generation of polyunsaturated fatty acids (PUFAs) related acyl-CoA esters. One pathway links PUFA, particularly arachidonic acid (AA) or adrenic acid, to CoA, producing PUFA-CoAs, which are subsequently converted to phosphatidylethanolamine (PE) via LPCAT3 [36]. The other pathway involves the production of fatty acid cholesterol esters by sterol O-acyltransferase 1 (SOAT1) in solute carrier family 47 member 1 (SLC47A1, also known as MATE1)-deficient human pancreatic ductal adenocarcinoma (PDAC) cells and NALM-6 leukemia cells, enhancing ferroptosis sensitivity [37]. While monocyte to macrophage differentiation-associated (MMD) activity can engage with ACSL4 and membrane-bound O-acyltransferase domain-containing 7 (MBOAT7), enhancing the influx of arachidonic acid into phosphatidylinositol and augmenting the sensitivity of OVCAR-8 and 786-O cells to ferroptosis, the broader impact of MMD on ferroptosis in other cancer cell lines, non-cancerous environments, and in vivo settings remains unexplored [38]. In contrast, LPCAT1 enhances membrane phospholipid saturation while reducing membrane PUFA levels through both endogenous and exogenous saturated fatty acid (SFA)-containing phospholipids [39]. This protective mechanism shields various cancer cell lines as well as mouse tumors, from peroxidation-induced membrane damage, thereby inhibiting ferroptosis [39]. Furthermore, the direct potential of arachidonic acid-phosphatidylinositol species in promoting ferroptosis formation independent of arachidonic acid-phosphatidylethanolamine and arachidonic acid-phosphatidylcholine phospholipids warrants further investigation [38]. Moreover, acetyl-CoA carboxylase alpha (ACACA, also known as ACC1) potentially contributes to the elongation of PUFAs, facilitating FIN56-induced degradation of GPX4 protein and the induction of ferroptosis in HT-1080 cells [40].

The lipoxygenase (ALOX) family, comprising multiple isoforms such as ALOX5, ALOX12, ALOX15, among others, facilitates the oxygenation of PUFAs, such as arachidonic acid. This enzymatic activity leads to the generation of various bioactive lipid mediators, which can induce ferroptosis in a manner contingent upon the cell type [41, 42]. This process can be augmented by phosphatidylethanolamine binding protein 1 (PEBP1), which complexes with ALOX15 to boost the production of lipid peroxides [43]. Diminished PEBP1 expression correlates with cancer progression and has been proposed as a metastasis suppressor in select contexts by impeding epithelial-mesenchymal transition and the metastatic dissemination process in breast cancer cells [44]. Targeting ALOX15 has proven effective in mitigating injuries induced by multiple modes in mice [43]. However, depleting *Alox15* failed to rescue acute renal failure induced by *Gpx4* depletion [45]. ALOX12 is essential for TP53-mediated tumor suppression in various cancer cell lines (e.g., U2OS, H1299, and A549 cells) as well as in murine models [46]. This effect is independent of ACSL4 [46]. Similarly, pleckstrin homology like domain family A member 2 (PHLDA2) forms a complex-mediated phosphatidic acid peroxidation with ALOX12, independent of the ACSL4-GPX4 pathway, and is capable of suppressing tumors in both immunodeficient and immunocompetent mice [47]. Thus, each ALOX isoform may have distinct roles and regulatory mechanisms in ferroptosis, adding complexity to deciphering their specific contributions.

ALOX-independent pathways, such as cytochrome P450 oxidoreductase (POR), also contribute to promoting lipid peroxidation and subsequent ferroptosis [20, 21]. POR, primarily expressed in the endoplasmic reticulum (ER), can initiate phospholipid peroxidation and ferroptosis by generating lipid hydroperoxides and 4HNE, independently of the cytochrome P450 family [20, 21]. Unlike the tissue-specific expression of the ALOX family, POR exhibits widespread expression throughout various tissues. Therefore, caution and specificity are necessary when targeting POR to inhibit ferroptosis [48]. Furthermore, prostaglandin-endoperoxide synthase (PTGS) enzymes, such as prostaglandin E2 (PGE2), play context-dependent roles in ferroptosis [49, 50]. A recent study suggests that PGE2 can induce ferroptosis specifically in CD8^+^ T cells, rather than other forms of cell death, by inhibiting the mechanistic target of rapamycin kinase (MTOR) pathway [51]. This inhibition, in turn, restricts IL2-mediated antitumor activity [51]. Understanding the interplay between these pathways is crucial for developing targeted therapeutic strategies against diseases associated with dysregulated ferroptosis mechanisms.

#### Lipids

Lipids represent a diverse array of molecules characterized by varying chemical structures. Among these, certain lipids exhibit the capacity to inhibit PUFA-related lipid peroxidation through competitive mechanisms or by serving as antioxidants. For example, ACSL3-dependent production of monounsaturated fatty acids (MUFAs) inhibits lipid peroxidation and ferroptosis in cancer cells, potentially by directly displacing PUFAs from phospholipids in cell membranes [42, 52]. Mitochondrial glutamate transporter solute carrier family 25 member 22 (SLC25A22), which not only promotes NAPDH-mediated glutathione (GSH; an antioxidant) production, but also promotes the expression of stearoyl-CoA desaturase (SCD, also known as SCD1) in human PDAC cells, produces MUFAs to inhibit ferroptosis [53]. Likewise, exogenous oleic acid attenuates acute iron overload-induced injury in Hep G2 and SK-N-Be(2) cells, *Caenorhabditis elegans*, and murine models by reducing the levels of PUFA acylphospholipids and ether-bonded phospholipids [54]. However, further investigation is warranted to elucidate the impact of oleic acid’s protective effect in mice, considering that iron overload in humans typically manifests as a chronic condition [54].

Another regulator of MUFA production involves MBOAT1 and MBOAT2, which are upregulated by sex hormone receptors [55]. These enzymes inhibit ferroptosis in cancer cells by restructuring the cellular phospholipid profile to generate phospholipids containing MUFAs [55]. Furthermore, SFAs dependent on LPCAT1 have been shown to inhibit ferroptosis [39]. These findings suggest a competitive relationship among PUFAs, MUFAs, and SFAs in shaping lipid composition to regulate ferroptosis activity [13]. The cyclin dependent kinase inhibitor 2A (CDKN2A, also known as p16 or p19), a tumor suppressor, is essential for regulating the G1 to S phase transition in the cell cycle, controlling cell growth. Interestingly, *CDKN2A* deletion alters lipid metabolism, enhancing ferroptosis sensitivity in glioblastoma [56]. Similarly, stabilization of TP53 and inhibition of cyclin-dependent kinase 4/6 (CDK4/6) activity induced cell cycle arrest and heightened sensitivity to covalent GPX4 inhibitor (GPX4i)-induced ferroptosis, while exhibiting no effect on systemic xc^−^ inhibitors, in HT-1080N, Caki-1N, H1299N, T98GN, and A375 cells, as well as in vivo models [57]. Conversely, cell cycle arrest in human renal cell carcinoma cell lines (Caki-1, ACHN, 786-O, and TK10) attenuates ferroptosis by elevating lipid droplet content, a phenomenon dependent on diacylglycerol acyltransferases (DGATs). Targeting DGATs reverses cell cycle-induced resistance to ferroptosis [58], indicating variability in ferroptosis susceptibility across different cell cycle stages.

#### Cholesterol

Several endogenous lipid metabolites exhibit direct antiferroptotic activity by limiting lipid peroxidation. For instance, 7-dehydrocholesterol, a precursor of cholesterol, acts as a radical-trapping antioxidant capable of suppressing ferroptosis, thereby presenting a novel metabolic target for improving ferroptosis-related cancer therapy [59, 60]. Specifically, the enzyme sterol-C5-desaturase (SC5D) within the cholesterol synthesis pathway promotes the production of 7-dehydrocholesterol, which inhibits ferroptosis due to the structural properties conferred by its 5,7-unsaturated double bond. This double bond directly impedes phospholipid peroxidation. Conversely, 7-dehydrocholesterol reductase (DHCR7), responsible for converting 7-dehydrocholesterol to cholesterol, promotes ferroptosis. Thus, modulation of 7-dehydrocholesterol levels can inhibit tumor growth or prevent ischemia–reperfusion kidney injury in mice [61, 62]. Moreover, cholesterol present in the tumor microenvironment induces the upregulation of CD36 expression in mouse melanoma B16 and mouse multiple myeloma tumor-infiltrating CD8^+^ T cells, facilitating fatty acid (such as arachidonic acid) uptake [63]. This process leads to lipid peroxidation, thereby compromising antitumor immunity in a murine melanoma model achieved by inoculating B16 cells into the syngeneic C57BL/6 J mouse strain [63]. Long-surviving melanoma patients and multiple myeloma patients exhibit reduced CD36 expression on CD8^+^ T cells and demonstrate responsiveness to programmed cell death 1 (PDCD1, also known as PD-1) treatment. CD8^+^ T cells infiltrating mouse melanoma B16 or colorectal MC38 tumors uptake oxidized low-density lipoprotein (OxLDL) in a CD36-dependent manner, consequently triggering lipid peroxidation. This leads to downstream activation of mitogen-activated protein kinase 14 (MAPK14; also known as p38) kinase and inhibition of the antitumor effector function of CD8^+^ T cells [64]. One limitation of this study is that it does not address how CD36 selectively uptakes different lipids to promote cell survival or induce cell death. Additionally, the degradation of lipid droplets through lipophagy contributes to providing substrates for lipid peroxidation, thereby promoting ferroptosis in hepatocellular carcinoma cells both in vitro and in xenograft mouse models [65]. Conversely, enhancing tumor protein D52 (TPD52)-dependent lipid storage in lipid droplets restricts ferroptosis [65], suggesting the pivotal role of dynamic lipid droplet levels in regulating ferroptosis sensitivity.

Collectively, the presence of lipid peroxidation in ferroptosis suggests a complex interplay among various lipids, enzymes, and organelles in diverse cellular contexts, indicating that it is not solely determined by a single factor. Efforts are directed towards identifying and characterizing molecules that regulate lipid peroxidation in ferroptosis. This encompasses the study of both endogenous regulators within cells and exogenous factors that modulate lipid peroxidation susceptibility.

### Iron accumulation

Iron is an essential micronutrient crucial for various cellular processes, including cell proliferation and death. In the context of ferroptosis, iron accumulation participates not only in the direct peroxidation of PUFA containing phospholipids through the Fenton reaction but also serves as a cofactor for specific enzymes (e.g., ALOXs and POR), thus playing a pivotal role in initiating ferroptosis [42, 66].

#### Intracellular iron homeostasis regulation

Manipulation of intracellular iron homeostasis can directly influence ferroptosis sensitivity, involving processes such as iron uptake, utilization, storage, and export. Typically, iron, primarily in the form of Fe^3+^, binds to transferrin (TF), enters the cell via the transferrin receptor (TFRC, also known as CD71) [67], and is transported to the endosome where it is reduced to Fe^2+^ by STEAP3 metalloreductase (STEAP3) before being released into the cytosol [68]. This reactive Fe^2+^ preferentially forms various iron-binding complexes (such as ferritin and iron-sulfur clusters), participating in a variety of physiological and biochemical reactions [69, 70]. Excess Fe^2+^ is excreted from the cell via solute carrier family 40 member 1 (SLC40A1, also known as ferroportin 1) to produce Fe^3+^ [71]. Dysregulation at any step may affect ferroptosis sensitivity by increasing the labile iron pool content in the cytoplasm.

#### Labile iron pool

Modulation of the labile iron pool capacity to induce ferroptosis can occur through several major mechanisms: (1) Induction of TFRC or solute carrier family 39 member 14 (SLC39A14) expression augments exogenous iron uptake, promoting the induction of ferroptosis in liver cells or during coxsackievirus B3 infection in HeLa cells [72, 73]. Conversely, heat shock protein family B (small) member 1 (HSPB1, also known as HSP25 or HSP27) inhibits iron uptake by remodeling the cell cytoskeleton, thereby impeding ferroptosis in human cancer cells (e.g., HeLa, U2OS, and LNCaP) [74]. (2) Aconitase 1 (ACO1) or iron-responsive element binding protein 2 (IREB2) mediates the utilization of free iron, leading to the promotion of ferritin formation. ZFP3 ring finger protein (ZFP3, also known as tristetraprolin) maintains cellular iron pool capacity during iron deficiency in various cancer cells by degrading mRNA transcripts, thereby reducing the synthesis of iron-binding proteins, particularly iron-sulfur proteins [75, 76]. (3) Nuclear receptor coactivator 4 (NCOA4)-dependent ferritinophagy facilitates ferritin degradation, resulting in the release of free iron and promoting the induction of ferroptosis in pancreatic cancer cells [77]. Conversely, glutamic-oxaloacetic transaminase 1 (GOT1) inhibits ferritinophagy-mediated ferroptosis in pancreatic cancer cells [78]. (4) Decreased expression of iron-exporting proteins, such as SLC40A1 and prominin2 (PROM2), either through transcriptional regulation or protein degradation mechanisms, can lead to iron accumulation and subsequent ferroptosis in various cancer cells [71, 79].

Additionally, organelles such as lysosomes, Golgi, and mitochondria serve as important sources of labile iron [27]. Consequently, the regulation of iron homeostasis is multifaceted and involves not only the expression of specific pathway proteins but also communication between organelles. As iron plays a pivotal role in numerous cellular processes such as DNA synthesis, energy metabolism, and oxygen transport, achieving modulation of iron metabolism to induce ferroptosis without compromising these vital functions requires a comprehensive understanding of the molecular mechanisms at play.

### Membrane rupture

In the context of ferroptosis, the occurrence of plasma membrane lipid peroxidation and rupture typically constitutes a late event. Initially, lipid peroxidation manifests in the ER membrane following the initiation of ferroptosis, and subsequently extends to other organelle membranes and eventually the plasma membrane [80]. Ferroptotic cells not only undergo targeted removal but also transmit signals to neighboring healthy cells. For instance, treatment of primary mouse embryonic fibroblasts (MEFs) and NIH3T3 cells with ferroptosis inducers (e.g., erastin and RSL3) and subsequent collection of drug-free supernatants, followed by co-culture with healthy cells, leads to reduced senescence-associated β-galactosidase-positive cells and the induction of ferroptosis through an autophagy-dependent mechanism [81]. Utilizing the indicator SYTOX Green has revealed that the time difference between neighboring cell deaths, compared to the mean distribution of randomly generated death times, indicates that inducers like erastin diffuse in a wave-like manner in neighboring cells, dependent on iron and lipid peroxidation. This diffusion necessitates the formation of plasma membrane pores to release the contents of substances such as calcium [82]. In contrast, ML162 does not propagate ferroptosis signals to neighboring cells [82], indicating that different GPX4 inhibitors may have varying effects on the induction of ferroptosis in tissues. Furthermore, in a zebrafish model, topical application of arachidonic acid and skin-permeable peroxides to uninjured larval fins induced low levels of lipid peroxidation similar to that seen in wounds [83]. Rapid arachidonic acid attraction to leukocytes via dual oxidase and ALOX5 indicates a role for lipid peroxidation as a spatial redox relay [83].

Although the molecular mediator responsible for the final steps of membrane rupture in ferroptosis remains poorly understood, one hypothesis involves the involvement of ninjurin 1 (NINJ1) protein [84]. NINJ1 is a cell adhesion molecule that plays various roles in cellular processes, including cell adhesion, migration, and signaling. Knockout of *Ninj1* in BMDMs and MEFs inhibits ferroptosis induced by RSL3 and ML162 [84]. Mechanistically, the activation of NINJ1 occurs downstream of lipid peroxidation and calcium influx, leading to the insertion of its originally extracellular α-helices into the cell membrane, thereby forming larger aggregates [84]. Deletion of the α-helices inhibits pore formation, even if the cell undergoes death [85–87]. In a liver injury animal model, application of anti-NINJ1 antibody limited tissue damage, suggesting potential therapeutic avenues targeting membrane damage [87]. Of note, a separate investigation employing *Ninj1*^*−/−*^ RAW264.7 cells demonstrated that NINJ1 is dispensable for RSL3-induced ferroptosis [88]. In addition, NINJ1 can promote solute carrier family 7 member 11 (SLC7A11) degradation, thereby limiting the production of GSH and coenzyme A, and increasing erastin-induced ferroptosis sensitivity in HT-1080 cells [89]. However, there was no difference observed between wild-type and *NINJ1*^*−/−*^ HT-1080 cells in terms of sensitivity to RSL3 [89]. Thus, NINJ1 demonstrates a cell type- and stimulus-specific influence on the regulation of ferroptosis.

Endosomal sorting complex required for transport III (ESCRT-III) is a highly conserved protein complex with diverse roles in cellular processes, including endosomal sorting, membrane remodeling, and cytokinesis. Among its functions, ESCRT-III mediates the repair of damaged plasma membranes during lytic cell death and contributes to therapy resistance in cancer cells [90]. Comprising several protein subunits, including members of the charged multivesicular body protein (CHMP) family, such as CHMP2A, CHMP2B, CHMP3, CHMP4A, CHMP4B, CHMP4C, and CHMP6, ESCRT-III assembles into spiral filaments at sites of membrane damage. These subunits facilitate membrane scission and sealing, essential for membrane repair in HT-1080 and PANC-1 cells during ferroptosis [91, 92].

In summary, the propagation of lipid peroxidation signals in ferroptosis plasma membranes elucidates the mechanisms by which cell death signals spread to neighboring healthy cells or tissues through NINJ1-dependent or -independent pathways. In contrast, the activation of membrane repair mechanisms, such as ESCRT-III, presents promising avenues for therapeutic intervention to inhibit ferroptosis and mitigate tissue damage.

## Antioxidant systems in ferroptosis

### GPX4-dependent pathway

Organisms deploy a diverse array of antioxidant systems to counteract and rectify oxidative damage associated with ferroptosis. Among these systems, the selenoprotein GPX4 stands out as a central player in inhibiting ferroptosis across various cellular contexts and tissues. GPX4 exists in three isoforms: cytosolic (cGPX4), mitochondrial (mGPX4), and nuclear (nGPX4), each exhibiting distinct spatiotemporal expression patterns during embryonic development and adulthood. While cytosolic GPX4 predominantly inhibits ferroptosis in many instances, mitochondrial GPX4 also participates in ferroptosis inhibition under specific conditions. GPX4, featuring an active-site selenocysteine (Sec46) along with seven other cysteine residues (Cys2, Cys10, Cys37, Cys66, Cys75, Cys107, and Cys148), all potentially reactive with electrophiles [93]. Global knockout of *Gpx4* results in embryonic lethality in mice [94], while conditional knockout of *Gpx4* can lead to spontaneous ferroptotic damage in certain mouse tissues, such as the kidney [45], indicating the key role of ferroptosis in development. Functionally, GPX4 utilizes GSH to reduce lipid hydroperoxides to lipid alcohols, thus averting lipid peroxidation and preserving cellular membrane integrity [95]. The interaction between creatine kinase B (CKB) and GPX4 facilitated the phosphorylation of GPX4 at S104, mitigated autophagic degradation of GPX4, and suppressed ferroptosis in hepatocellular carcinoma [96].

System xc^−^ consists of two key components: SLC7A11 (also known as XCT) and solute carrier family 3 member 2 (SLC3A2; also known as CD98 or 4F2hc). It functions as a cystine/glutamate antiporter responsible for importing cystine into the cell in exchange for glutamate. Cystine is subsequently reduced to cysteine, a crucial constituent of GSH. The classical ferroptosis inducers, erastin and RSL3, act as inhibitors of system xc^−^ and GPX4, respectively, underscoring the significance of the system xc^−^-GSH-GPX4 pathway in suppressing ferroptosis [9, 97, 98]. Due to its ubiquitous expression across various cell types, directly targeting GPX4 with covalent inhibitors may lead to significant adverse effects, potentially limiting the therapeutic utility of such inhibitors [99]. The transsulfuration pathway of methionine provides cancer cells with GSH to evade ferroptosis caused by cysteine deprivation. Conversely, intermittent dietary methionine deprivation significantly increases ferroptosis sensitivity in various tumor models [100].

GPX4 exhibits a multifaceted role not only in ferroptosis but also in other forms of cell death, such as apoptosis [101], necroptosis [102] and pyroptosis [103]. This versatility stems from GPX4’s involvement in various cellular homeostasis and stress response pathways. Similarly, genetic depletion or pharmacological inhibition (e.g., using HG106) of SLC7A11 can trigger non-ferroptotic cell death in *KRAS* mutant lung adenocarcinoma or melanoma [104, 105]. GSH can inhibit cuproptosis through its capacity to chelate copper [106]. These findings demonstrate the context-dependent role of the SLC7A11-GSH-GPX4 pathway in cell death. Similarly, using GSH depletion may not adequately reflect ferroptosis.

### GPX4-independent pathway

Among the increasingly reported GPX4-independent pathways, apoptosis inducing factor mitochondria associated 2 (AIFM2, also known as FSP1) emerges as a central regulator. Initially identified as a positive regulator of mitochondrial apoptosis, AIFM2 translocates from mitochondria to the plasma membrane in GPX4-low-expressing cancer cells when stimulated with ferroptosis inducers (e.g., RSL3 and ML162) [107, 108]. This translocation positions AIFM2 as a repressor of ferroptosis through an integrated mechanism, involving the production of reduced coenzyme Q10 (CoQH_2_) [107, 108] and reduced vitamin K (VKH_2_) [109, 110], as well as mediating ESCRT-III-dependent membrane repairs [111]. Vitamin K epoxide reductase complex subunit 1 like 1 (VKORC1L1) generates a reduced form of vitamin K that counteracts phospholipid peroxides and protects human pancreatic and melanoma cells from ferroptosis response independent of the GSH-GPX4 pathway [112]. AIFM2’s function in ferroptosis is reliant on phase separation, initiated by N-terminal myristoylation at S187, L217, and Q319, facilitated by the compound icFSP1 [110]. Furthermore, structural analysis revealed that AIFM2 binds to CoQ_10_ and flavin-adenine dinucleotide (FAD), facilitating catalytic activity by utilizing its carboxyl terminus to form a homodimer. The intermediate metabolite, 6-OH-FAD, serves as an active cofactor for AIFM2 and acts as an antioxidant, directly eliminating lipid peroxidation [113]. These discoveries highlight the location-specific role of AIFM2 in apoptosis and ferroptosis, along with its enzymatic ability to produce antioxidants. However, a crucial question remains: how does AIFM2 coordinate the dynamic response to ferroptotic damage, and what signals govern its activation of distinct downstream pathways?

Superoxide dismutase 2 (SOD2, also MnSOD) serves as a vital antioxidant enzyme primarily localized within the mitochondria. Its key function involves detoxifying the O_2_^−^ by converting it into O_2_ and H_2_O_2_. H_2_O_2_ not only induces mitochondrial apoptosis but also triggers ferroptosis, highlighting the need for distinct downstream pathways to mediate different RCD mechanisms [20]. Depletion of *SOD2* enhances the radiosensitivity of nasopharyngeal carcinoma cells by inducing ferroptosis [114]. The increased sensitivity to ferroptosis in *SOD2* knockdown cells can be reversed by dihydroorotate dehydrogenase (quinone) (DHODH) [114], a mitochondrial enzyme involved in pyrimidine biosynthesis essential for DNA and RNA synthesis. Although subject to debate, DHODH has been implicated in utilizing CoQ_10_ to produce CoQH_2_ within mitochondria, acting as a radical-trapping antioxidant to suppress lipid peroxidation and ferroptosis [115]. Furthermore, presenilin-associated rhomboid like (PARL)-mediated cleavage of StAR-related lipid transfer domain-containing 7 (STARD7) is necessary for the synthesis and translocation of CoQ_10_ from mitochondria to the plasma membrane, where it generates CoQH_2_ to inhibit ferroptosis [116]. While these studies shed light on mitochondrial defense mechanisms involving CoQ_10_ metabolism and transport, the regulation of CoQ_10_ levels across different organelle pools to balance ferroptosis responses remains elusive. Another mitochondrial defense mechanism against ferroptosis involves pyruvate dehydrogenase kinase 4 (PDK4), which inhibits pyruvate dehydrogenase-dependent pyruvate oxidation in pancreatic cancer cells [117].

In addition to AIFM2 and DHODH, an increasing number of GPX4-independent antioxidant enzymes have been identified, each playing a contextually significant role in inhibiting ferroptosis. GTP cyclohydrolase 1 (GCH1) participates in the biosynthesis of tetrahydrobiopterin (BH_4_), a vital cofactor for enzymes involved in neurotransmitter synthesis and nitric oxide production. BH_4_ contributes to cellular redox balance and ferroptosis inhibition [118]. Nitric oxide synthase 2 (NOS2, also known as iNOS) catalyzes nitric oxide production from L-arginine and oxygen, with its induction being triggered by various stimuli. In macrophages, NOS2 suppresses ferroptosis by inhibiting ALOX15-mediated lipid peroxidation, linking NOS2 induction to ferroptotic inflammation inhibition [119]. YEATS domain-containing 4 (YEATS4; also known as GAS41) interacts with NFE2 like BZIP transcription factor 2 (NFE2L2, also known as NRF2) and modulates its transcriptional activity through H3K27 acetylation (H3K27-ac), thereby selectively activating the transcription of NFE2L2 target genes to suppress ferroptosis in human melanoma and lung cancer cells [120]. NFE2L2 serves as a master transcription factor in oxidative stress responses. Besides regulating GPX4, SLC7A11, and AIFM2 expression, NFE2L2 upregulates microsomal glutathione S-transferase 1 (MGST1), aiding cellular detoxification in pancreatic cancer cells against ferroptotic activators [121]. MGST1, belonging to the glutathione S-transferase (GST) family, aids in detoxification by conjugating GSH to electrophilic compounds. Another GST family member, glutathione S-Transferase zeta 1 (GSTZ1), inhibits ferroptosis in bladder cancer cells [122]. Thioredoxin reductase 1 (TXNRD1) catalyzes thioredoxin reduction by utilizing NADPH as a cofactor and collaborates with GPX4 to maintain redox homeostasis [123, 124]. Conversely, malic enzyme 1 (ME1) serves as a supplier of cytoplasmic NADPH, exerting its role as a liver-specific ferroptosis inhibitor owing to its antioxidant properties [125]. Given NADPH’s role in GSH and TXN antioxidant systems, targeting ME1 is a promising strategy to increase sensitivity to ferroptosis. Peroxiredoxins (PRDX) reduce peroxides, protecting against oxidative stress. Both TXNRD1 and PRDX have context-dependent roles in inhibiting ferroptosis [123, 126, 127]. Additionally, Ca^2+^-independent phospholipase A2 group VI (PLA2G6, also known as iPLA2β or PNPLA9) plays a crucial role in membrane phospholipid metabolism, inhibiting ferroptosis in various cell types [128, 129]. Glutathione S-transferase Pi 1 (GSTP1) also facilitates the conjugation of GSH to 4-hydroxynonenal, thereby detoxifying lipid hydroperoxides independently of selenium glutathione peroxidase activity [130].

In conclusion, the human body has multiple antioxidant systems that collaboratively operate to mitigate ferroptotic oxidative stress and maintain cellular homeostasis [131]. Should one antioxidant system become overwhelmed or compromised, others can step in to compensate and offer protection against oxidative damage during ferroptosis.

## Regulation of ferroptosis

**Fig. 2:**
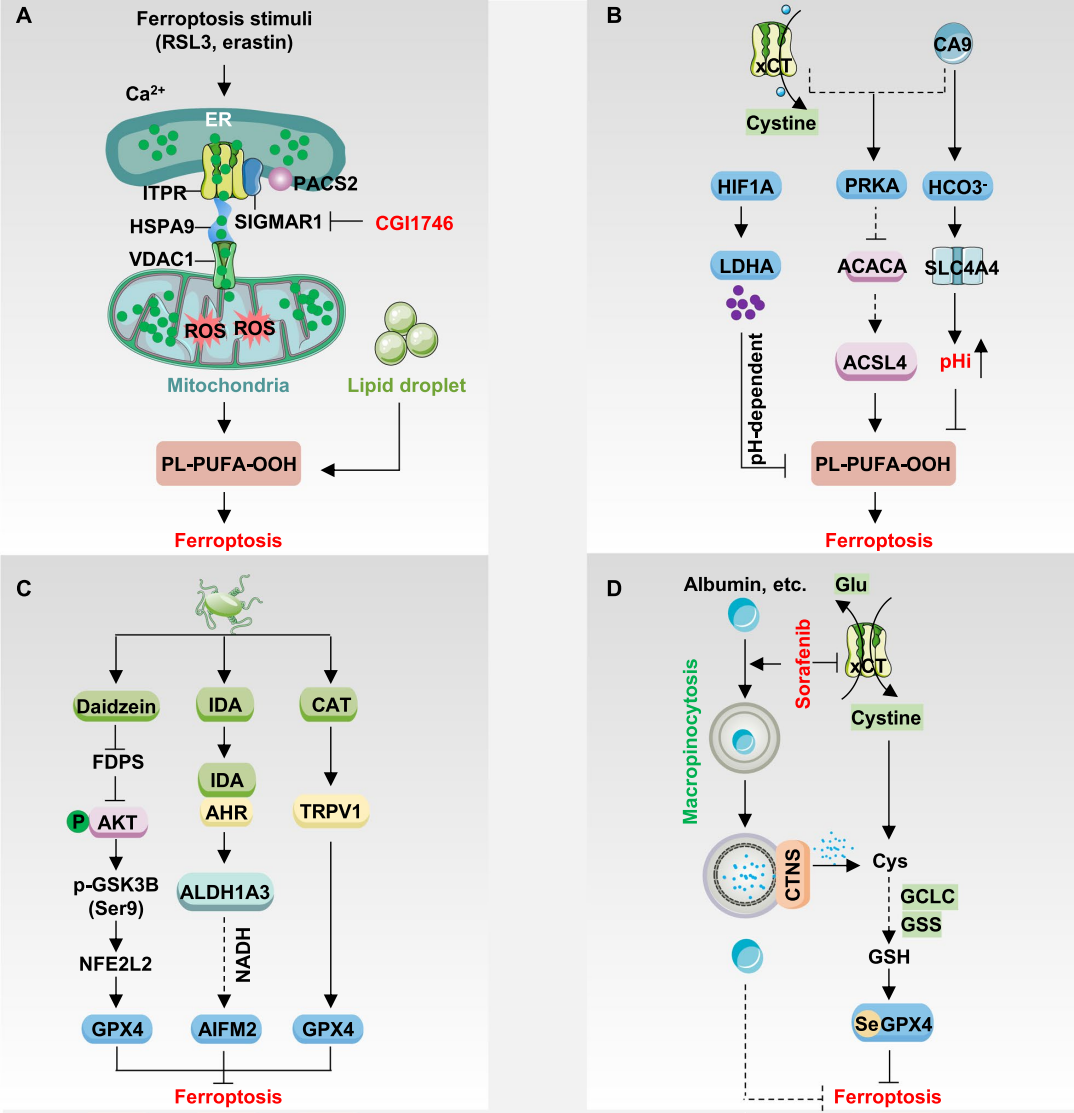
Regulation of ferroptosis. **A** SIGMAR1 interacts with ITPR to facilitate calcium exchange between the endoplasmic reticulum (ER) and mitochondria, promote lipid droplet catabolism, and enhance sensitivity to ferroptosis. Moreover, the PACS2, HSPA9/VDAC1 complex also mediates the transmission of ferroptosis signals from the ER to mitochondria. **B** CA9 inhibits ferroptosis through the AMPK pathway or by directly inducing alkalinization of intracellular pH. Additionally, HIF1A-dependent lactate accumulation inhibits ferroptosis via a pH-dependent mechanism. **C** Intestinal flora secretes metabolites such as IDA, CAT, or daidzein to modulate the expression of AIFM2 or GPX4 and suppress ferroptosis. **D** Cancer cells utilize the macropinocytosis pathway to uptake proteins like extracellular albumin to supplement cysteine and inhibit ferroptosis under conditions of system xc- inhibition. Moreover, albumin may directly inhibit lipid peroxidation

Cellular homeostasis is governed by numerous signaling networks, suggesting that processes regulating iron metabolism, redox balance, and cellular metabolism can profoundly impact ferroptosis. Several recent comprehensive reviews have extensively outlined the various signaling pathways involved in regulating ferroptosis [22, 132–135]. In this section, we explore four emerging regulatory mechanisms: membrane contact sites, hypoxia-dependent pH changes, interactions with intestinal flora, and pathways involving macropinocytosis (Fig. 2), which have received comparatively less attention in previous reviews.

### Membrane contact sites

Membrane contact sites are specialized regions where two organelles in a cell come into close proximity, allowing for direct communication and exchange of materials between them. These contact sites are formed by tethering proteins that bridge the membranes of the two organelles. Membrane contact sites play a role in various cellular processes, including lipid metabolism, calcium signaling, organelle dynamics, and membrane trafficking [136–139]. They play a crucial role in modulating homeostasis by enabling the exchange of essential molecules and signals implicated in cell survival and death pathways [140, 141].

Mitochondria-associated membranes (MAMs) are specialized regions where the membranes of the ER come into close proximity with the outer membrane of mitochondria. These contact sites allow for direct communication and functional interaction between the ER and mitochondria. In addition to regulating autophagy and apoptosis in a context-dependent manner [142, 143], a recent study has provided the first evidence that increased MAMs promote sensitivity to erastin, RSL3, or ML210-induced ferroptosis in multiple cancer cell lines, such as MDA-MB-468 and NCI-H1299, through enhanced calcium release from the ER into mitochondria (Fig. 2A) [144]. Under conditions of ferroptotic stress, there is an augmentation in the interaction between sigma non-opioid intracellular receptor 1 (SIGMAR1, also known as σ1R), a molecular chaperone situated in the MAMs, and inositol 1,4,5-trisphosphate receptor (ITPR, also known as IP3R). This enhancement prompts an exchange of calcium ions from the ER to mitochondria, thereby intensifying sensitivity to ferroptosis [144]. Conversely, targeting SIGMAR1 inhibits ferroptosis in cancer cells and mitigates cisplatin-induced acute kidney injury in mice through pharmacological means (using CGI1746 and BD1063) or genetic interventions [144]. SIGMAR1 is upregulated in several cancers, such as lung, breast, glioblastoma, esophageal, pancreatic, prostate, and liver cancer. It plays a role in promoting drug resistance and tumor progression [145–147]. Targeting SIGMAR1-mediated ferroptosis may present a novel strategy to shift cancer cells from a pro-survival to a pro-death state.

Furthermore, proteins or complexes that bridge the gap in the MAMs, such as the IP3R-heat shock protein family A (Hsp70) member 9 (HSPA9, also known as GRP75)-voltage dependent anion channel 1 (VDAC1) complex, along with other tethering factors like mitofusin 2 (MFN2), phosphofurin acidic cluster sorting protein 2 (PACS2), VAMP associated protein B and C (VAPB), and regulator of microtubule dynamics 3 (RMDN3, also known as PTPIP51), establish a platform for the transmission of ferroptosis signals from the ER to mitochondria. Mechanistically, SIGMAR1 inhibition diminishes ER-to-mitochondrial calcium transport, subsequent mitochondrial ROS production, and elevates intracellular PUFA-containing diacylglycerol levels [144]. These findings establish a direct connection between the ER and mitochondria mediated by calcium signals, with the ER acting as the initiator and mitochondria as the effector of ferroptosis. Hence, promoting the formation of MAMs may serve as a strategy to enhance ferroptosis-based antitumor therapy.

However, it’s still uncertain whether other membrane contact sites play a role in regulating ferroptosis. The interaction between the ER and the plasma membrane is essential for exchanging membrane components, especially lipids and proteins [148]. Grasping the significance of the ER-plasma membrane contact site in membrane transport, both vesicular and non-vesicular trafficking, and its potential impact on ferroptosis presents a notable challenge. Furthermore, calcium ions play pivotal roles in normal cellular functions such as muscle contraction, neurotransmitter release, and enzyme activation. Disrupting calcium signaling in cancer cells might inadvertently impact these vital functions in normal cells, possibly resulting in undesired side effects.

### Hypoxia-dependent pH changes

Regional hypoxia in tumors arises when oxygen concentration within certain areas of a tumor mass falls significantly below that of surrounding tissues. This phenomenon occurs due to the rapid proliferation of tumor cells outpacing the development of new blood vessels, leading to insufficient oxygen delivery to specific regions within the tumor. Consequently, hypoxic regions become a characteristic feature of many solid tumors [149].

In response to hypoxia, tumor cells undergo genetic and metabolic adaptations to survive and proliferate in low-oxygen environments [150–152]. Hypoxic conditions induce genetic changes in tumor cells, favoring the selection of aggressive phenotypes better suited to thrive under such conditions. Additionally, tumor cells in hypoxic regions undergo metabolic shifts, such as increased aerobic glycolysis (known as the Warburg effect), to generate energy and accumulate acidic byproducts. To counteract this acidic stress, cancer cells express pH-regulating enzymes and transporters to maintain an alkaline intracellular pH, supporting cell proliferation and survival [153].

The reversal of the pH gradient within cancer cells triggers metabolic reprogramming, and ferroptosis is linked to cellular metabolic status. Consequently, hypoxia-dependent pH regulation emerges as a critical pathway in governing ferroptosis (Fig. 2B). For instance, carbonic anhydrase 9 (CA9)-mediated intracellular alkaline pH inhibits ROS production. Targeting CA9 enhances ferroptosis driven by NFS1 cysteine desulfurase (NFS1) inhibition in a pH-dependent manner in human breast cancer cells [154]. Mechanistically, CA9 inhibition or interference with sodium-driven bicarbonate transport, coupled with targeting of SLC7A11, leads to decreased activation of protein kinase AMP-activated catalytic subunit (PRKA; also known as AMPK), increased ACACA activation, and enhanced ACSL4 expression [154]. In contrast, under hypoxic conditions, hypoxia-inducible factor 1 subunit alpha (HIF1A, also known as HIF1α) promotes ferroptosis resistance in several cancer cell lines. This resistance is mediated by the upregulation of solute carrier family 1 member 1 (SLC1A1), enhancing glutamate-cystine transport efficiency, and lactate dehydrogenase A (LDHA)-mediated lactate accumulation in a pH-dependent manner, which runs parallel to the SLC7A11 and AIFM2 systems [155]. Unlike the acidic environment induced by hydrochloric acid (HCl), acidic conditions induced by HCl (pH 6.6) promote ferroptosis induced by erastin and RSL3 under both normoxia and hypoxia. Inhibition of HIF1A sensitizes mouse solid tumors to ferroptosis inducers [155]. These findings generally support the notion that hypoxia-dependent pH changes inhibit ferroptosis.

The HIF family consists of heterodimeric proteins composed of an oxygen-sensitive alpha subunit (HIF1A, endothelial PAS domain protein 1 [EPAS1, also known as HIF2α], or HIF3A [also known as HIF3α]) and a constitutively expressed beta subunit aryl hydrocarbon receptor nuclear translocator (ARNT, also known as HIF1β). However, the HIF family is commonly up-regulated in human cancers, demonstrating a dual role in regulating ferroptosis by modulating various target genes [14, 156–159]. For instance, the obesity-dependent adipokine chemerin regulated by EPAS1 inhibits fatty acid oxidation, allowing clear cell kidney cancer to evade ferroptosis. This evidence highlights the complex mechanism through which hypoxia impacts ferroptosis. Hence, it is essential to elucidate the primary pathways regulated by HIFs across various contexts to enable targeted interventions for ferroptosis.

Furthermore, tumor cells exhibit adaptive responses to fluctuations in pH by initiating compensatory mechanisms to preserve pH homeostasis. For instance, cancer cells may enhance the expression of pH-regulating proteins and transporters or modify metabolic pathways to offset alterations in extracellular pH levels. Targeting a single pH-regulating mechanism could trigger the activation of alternative pathways, thereby constraining the efficacy of pH-targeted therapies. A comprehensive understanding of the influence of pH on ferroptosis necessitates a thorough characterization of pH-regulating proteins and transporters across various stages of ferroptosis.

### Intestinal flora

Intestinal flora, also termed gut microbiota or gut microbiome, encompasses the diverse assemblage of microorganisms residing within the gastrointestinal tract, primarily colonizing the colon in both humans and other animal species. This microbial community comprises bacteria, viruses, fungi, and other microorganisms. The composition of intestinal flora exhibits significant inter-individual variability, influenced by factors, such as dietary habits, genetic makeup, age, and environmental exposures. Perturbations in gut microbiota composition have been associated with altered sensitivity to various forms of host cell death, including apoptosis [160], pyroptosis [161], and ferroptosis [162, 163].

As anticipated, impaired ferroptosis plays a role in infectious and sterile inflammatory diseases (Fig. 2C). For instance, protein tyrosine phosphatase A (PtpA), secreted by the tuberculosis (TB)-causing pathogen *Mycobacterium tuberculosis* (Mtb), instigates ferroptosis in immune cells, thereby fostering Mtb pathogenicity and dissemination [163]. Other pathogen-associated molecular patterns (PAMPs), such as lipopolysaccharide (LPS), can induce ferroptosis in immune and endothelial cells, thereby accelerating the inflammatory response [164, 165]. Conversely, the production of capsiate by gut microbes enhances GPX4 expression via the activation of transient receptor potential cation channel subfamily V member 1 (TRPV1), thereby inhibiting intestinal ischemia–reperfusion injury or hypoxia/reoxygenation-induced ferroptosis in mice [166]. Furthermore, *Lactobacillus vaginalis* β-galactosidase releases daidzein to inhibit farnesyl diphosphate synthase (FDPS), consequently activating the AKT serine/threonine kinase (AKT)-glycogen synthase kinase 3 beta (GSK3B)-NFE2L2 pathway-dependent GPX4 expression and mitigating ferroptosis-induced liver injury in mice [167]. Thus, understanding the microbiota’s compositions and metabolites affecting ferroptosis is crucial for grasping the intestinal flora’s dual role in this process.

Recent research has also highlighted the connection between intratumoral bacteria and cellular ferroptosis, impacting tumor development (Fig. 2C). Colorectal cancer patients often display dysbiosis, characterized by shifts in gut microbiota composition and function, including a reduction in beneficial bacteria and an increase in potentially harmful ones. A recent study suggests that anaerobic *Peptostreptococcus anaerobius* fosters colorectal carcinogenesis by producing the tryptophan metabolite, trans-3-indoleacrylic acid (IDA), which inhibits ferroptosis through activating the aryl hydrocarbon receptor (AHR) [162]. Specifically, AHR-dependent transcriptional upregulation of aldehyde dehydrogenase 1 family member A3 (ALDH1A3) uses retinaldehyde to generate NADH, further promoting AIFM2-mediated CoQ_10_H_2_ synthesis to inhibit ferroptosis [162]. Anaerobes are significantly enriched in colorectal cancer patients. IDA treatment or *P. anaerobius* implantation promotes colorectal cancer progression associated with decreased ferroptotic response in xenograft models and *Apc*^*Min/*+^ mice [162, 168].

These findings elucidate how gut microbiota contribute to colorectal cancer development by inhibiting ferroptosis. Additionally, the hepatitis B virus promotes the development of hepatocellular carcinoma by inhibiting ferroptosis [169]. These findings suggest that pathogens drive tumorigenesis through the suppression of ferroptosis. However, chronic ferroptotic damage can induce inflammation and promote tumor development in pancreatic and liver cancer models [170–172]. Besides anti-ferroptotic AHR ligands such as IDA, pro-ferroptotic AHR ligands, such as L-kynurenine, induces cell death in natural killer cells, fostering gastric tumor growth [173]. Additionally, tryptophan metabolites serotonin and 3-hydroxyanthranilic acid enable various tumor cells (e.g., HT-1080 and B16F10) to evade ferroptosis by reducing oxidized phospholipid levels through their radical-trapping antioxidant activity, both in vitro and in vivo [174]. These findings indicate that tryptophan metabolism exhibits a dual role in modulating ferroptosis, contingent upon the specific tumor type.

Collectively, the isolation and differentiation of specific intestinal flora and tumor-resident microbiota, along with their metabolic products, pose challenges; however, they hold promising applications [175–177].

### Macropinocytosis

A primary pathway for inducing ferroptosis in cancer cells involves nutrient deprivation of the amino acid cystine or the pharmacological inhibition of SLC7A11 [178]. Cystine is primarily acquired by cells directly from the extracellular space via system xc^−^. Additionally, cancer cells can uptake extracellular substances for nutrients through alternative pathways such as macropinocytosis, a non-selective endocytic process observed in various mammalian cells, including cancer cells and cancer-associated fibroblasts [179]. Consequently, when cancer cells undergo cystine deprivation-induced ferroptosis, they activate macropinocytosis to evade ferroptosis induction (Fig. 2D). For instance, HT-1080 cancer cells uptake extracellular albumin via macropinocytosis and release it into the cytoplasm after lysosomal breakdown to liberate cysteine/cystine for the synthesis of anti-ferroptotic metabolites such as GSH [180]. This protective mechanism is further potentiated by mechanistic target of rapamycin kinase 1 (MTORC1) inhibition, underscoring the significance of lysosomal activity. Indeed, albumin catabolism within the lysosome depends on cathepsin B (CTSB) and is facilitated by cystinosin, the lysosomal cystine transporter (CTNS) [180]. MTOR inhibition and albumin supplementation still confer protection against U2OS cells during lysosomal inactivation, suggesting a direct antioxidant effect of albumin [180]. Nevertheless, CTSB may also promote ferroptosis through lysosomal cell death induction [181], making direct lysosome targeting challenging for enhancing ferroptosis, as lysosomes serve not only as recycling organelles for amino acid synthesis and energy production but also as a pivotal hub for various cell death pathways [182].

Sorafenib, a first-line therapeutic agent for hepatocellular carcinoma, may induce ferroptosis by targeting system xc^−^ [183]. However, hepatocellular carcinoma can utilize macropinocytosis as an alternative cysteine acquisition pathway. Sorafenib treatment induces mitochondrial dysfunction in human hepatocellular carcinoma cells (SK-Hep1 and Huh7), activating the phosphatidylinositol-4,5-bisphosphate 3-kinase catalytic (PI3K)-Rac family small GTPase 1 (RAC1)-p21 (RAC1) activated kinase 1 (PAK1) pathway-dependent macropinocytosis, thereby imparting ferroptosis resistance [184]. RAS is pivotal for macropinocytosis formation [185], yet it also inhibits lipid peroxidation through multiple pathways such as NFE2L2 [104] and fatty acid synthase (FASN) [186], suggesting that targeting RAS may have varied effects on specific cancer cells.

In summary, macropinocytosis-dependent ferroptosis inhibition primarily involves extracellular substance uptake to supplement anti-ferroptosis metabolites. However, this protective mechanism may not be universal, as the macropinocytosis level in non-cancer cells is significantly lower than that in cancer cells [187, 188]. Furthermore, both macropinocytosis and autophagy can be modulated by similar signaling pathways, including those associated with growth factors, nutrient availability, and stress responses [189]. Distinguishing the roles of these pathways in regulating ferroptosis-related therapy across different cancer cell types is an intriguing area for further investigation.

## The pathological significance of ferroptosis

**Fig. 3:**
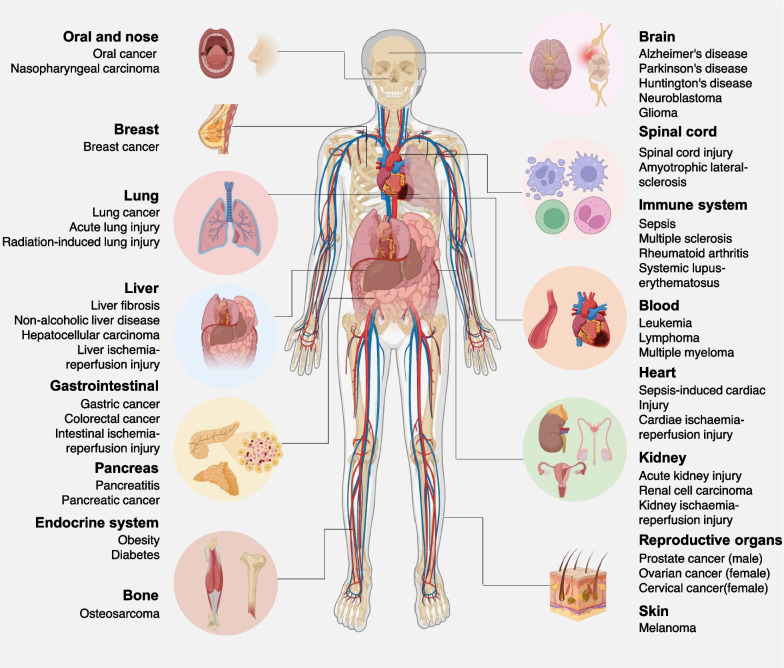
The pathological significance of ferroptosis. Ferroptosis has been implicated in a variety of diseases across different organs and tissues. In cancer, for example, ferroptosis resistance contributes to tumor progression and treatment resistance, while inducing ferroptosis has emerged as a potential therapeutic strategy. In neurodegenerative diseases such as Alzheimer’s and Parkinson’s, excessive lipid peroxidation and ferroptosis have been observed in affected brain regions, suggesting a role in neuronal death. Ischemia–reperfusion injury in organs like the heart and kidney involves oxidative stress and lipid peroxidation, leading to tissue damage characteristic of ferroptosis. Understanding the mechanisms and regulation of ferroptosis in various diseases holds promise for the development of novel therapeutic interventions targeting this pathway

In recent years, there has been growing recognition of ferroptosis as a contributing factor in the development of various diseases affecting nearly every organ system. This section will delve into the involvement of ferroptosis in conditions, such as cancer, neurodegeneration, sepsis, ischemia–reperfusion injury, autoimmune disorders, and metabolic disorders (Fig. 3). These diseases are highly susceptible to ferroptosis due to their elevated lipid content and the presence of iron, which catalyzes lipid peroxidation. Among the multiple signaling pathways, the impaired GPX4 pathway and increased ferritinophagy play central roles in mediating ferroptosis in these diseases.

### Cancer

Apoptosis has long been regarded as the primary mechanism for eliminating cancer cells. However, the dysregulation of apoptotic pathways, particularly the activation of anti-apoptotic mechanisms, often limits the efficacy of apoptosis-based cancer treatments [190, 191]. Over the past decade, extensive research into compounds targeting cancer-related genes and signaling pathways, particularly those associated with *KRAS* mutations, has unveiled a close association between ferroptosis and various cancers. Given the heightened metabolic activity and increased levels of ROS and iron content typically observed in cancer cells [192, 193], it is logical to consider cancer cells as potentially more susceptible to ferroptosis induction. Consequently, targeting ferroptosis represents a promising vulnerability in combating cancers (discussed later), including those exhibiting resistance to apoptosis, albeit through different mechanisms.

However, cancer cells have evolved various strategies to counteract the metabolic and oxidative stresses associated with ferroptosis. For instance, the stress-inducible nuclear protein 1, transcriptional regulator (NUPR1) promotes ferroptosis resistance in human PDAC cells and mouse models by upregulating lipocalin 2 (LCN2) expression, thereby reducing iron accumulation and subsequent oxidative damage [194]. PDK4-mediated inhibition of pyruvate oxidation and heat shock protein family A member 5 (HSPA5)-mediated stabilization of GPX4 have been identified as mechanisms for inhibiting ferroptosis in PDAC cells and mouse models [117, 195]. De novo pyrimidine biosynthesis involves cytosolic carbamoyl-phosphate synthetase II, aspartate transcarbamylase, and dihydroorotase, uridine monophosphate synthetase (UMPS), and DHODH, contributing to ferroptosis resistance in cancer cells (e.g., HeLa cells) and in xenograft models [196]. Similarly, increased expression of the cystine-glutamate antiporter SLC7A11 and the antioxidant transcription factor NFE2L2 serve as crucial pathways for mitigating ferroptosis [197, 198]. High expression of SLC7A11 under glucose starvation conditions may instead induce disulfidptosis [199], while NFE2L2 also regulates heme oxygenase 1 (HMOX1, also known as HO-1) to modulate ferroptosis [200]. Thus, the sensitivity or resistance of a particular cancer to ferroptosis induction depends on its unique genetic makeup.

In addition to its widespread use as a strategy to inhibit established tumor growth, several gene engineering models suggest that ferroptotic damage can promote tumorigenesis through damage-associated molecular pattern (DAMP)-mediated inflammation and subsequent immune suppression. For instance, depleting *Gpx4* in the pancreas or using high-iron diets in studies involving caerulein-induced pancreatitis and *Kras*^*G12D*^-induced pancreatic cancer in mice accelerated experimental pancreatitis and tumorigenesis [170, 201]. Ferroptotic pancreatic cells release oncogenic KRAS protein via exosomes, which are taken up by macrophages through the advanced glycosylation end-product specific receptor (AGER, also known as RAGE). This uptake induces a pro-tumor M2 polarization of macrophages [202]. Similarly, liver-specific knockout of *Gpx4* accelerates diethylnitrosamine-induced hepatocellular carcinoma by releasing high-mobility group box 1 (HMGB1), recruiting myeloid-derived suppressor cells (MDSCs), and upregulating the checkpoint protein CD274 (also known as PD-L1) [171]. In diethylnitrosamine-induced hepatocellular carcinoma mouse models, conditional deletion of activating transcription factor 4 (*Atf4*), an ER stress-related transcription factor, enhances ferroptotic liver damage by downregulating SLC7A11, thus promoting liver tumorigenesis [203].

Therefore, achieving a comprehensive understanding of the dual role of ferroptosis in various stages of cancer necessitates the assessment of cancer genomes as well as the tumor microenvironment, both of which are crucial internal and external factors in tumorigenesis [170, 204–207].

### Neurodegeneration

Neurodegeneration encompasses a spectrum of diseases, including Alzheimer’s disease, Parkinson’s disease, Huntington’s disease, and others, characterized by neuronal death and loss of neurological function [208]. Iron accumulation and lipid peroxidation are common pathophysiological features of these conditions. Glutamate-induced excitotoxicity is recognized as a key contributor to neurodegenerative diseases [209], and high extracellular glutamate levels can inhibit system xc^−^ and induce ferroptosis, implicating ferroptosis in the regulation of neurodegeneration. Indeed, ferrostatin-1, a ferroptosis inhibitor, blocks glutamate-induced neuronal excitotoxic death in an ex vivo model of rat hippocampal slice culture.

At the genetic level, depletion of *Gpx4* promotes neuronal damage and neurodegeneration in mice [210], while *Gpx4* overexpression prolongs survival and delays disease onset in amyotrophic lateral sclerosis models. Iron chelators and lipophilic radical-trapping antioxidants have shown promise in various in vitro experimental models of neurodegenerative diseases [211]. Additionally, ACSL4-dependent ferroptosis promotes experimental autoimmune encephalitis in mouse models by enhancing T cell activation. Inhibiting ferroptosis with ceruloplasmin improves the behavioral phenotype of experimental autoimmune encephalitis mice and prevents neuronal cell death [212]. Microglial ferroptosis mediated by SEC24 homolog B, COPII coat complex component (SEC24B) accelerates neurodegeneration in a human induced pluripotent stem cell-derived microglia model grown in a triple culture system [213].

In conclusion, various pathways involved in ferroptosis influence the progression of neurodegenerative diseases, making targeting ferroptosis a promising therapeutic approach. However, the systemic use of iron chelators and potential side effects of antioxidants require careful evaluation.

### Sepsis

Sepsis, a severe medical condition triggered by systemic inflammation in response to infection, often arises from bacterial, viral, or fungal pathogens. This immune response leads to widespread inflammation, clotting, impaired blood flow, organ dysfunction, and potentially life-threatening complications such as septic shock. Emerging evidence implicates ferroptosis in sepsis, as it directly exacerbates or promotes organ damage associated with this condition. For instance, solute carrier family 39 member 8 (SLC39A8)-driven ferroptosis is a primary contributor to monocyte loss in sepsis patients, resulting in immunosuppression, while inhibiting SLC39A8 can reduce LPS-induced lipid peroxidation [214]. In murine models of LPS-induced sepsis, intravenously administered melanin nanoparticles attenuate myocardial injury by inhibiting ferroptosis, thereby reducing oxidative stress, inflammation, and maintaining mitochondrial homeostasis [215].

In bacterial infection-mediated sepsis, GPX4 negatively regulates sepsis severity in macrophages and lethality in mice. Conditional knockdown of *Gpx4* in myeloid cell lines increases lipid peroxidation-dependent caspase-11 activation and gasdermin D (GSDMD) cleavage, triggering macrophage pyroptosis via a phospholipase C gamma 1 (PLCG1)-dependent pathway [103]. The administration of the antioxidant vitamin E, rather than liproxstatin-1, prevents polymicrobial sepsis in *Gpx4* conditional knockout mice in myeloid cells, suggesting that ferroptosis is not required for *Gpx4* depletion-induced cell death in myeloid cells responding to bacterial infections [103]. Particularly, the efficacy of vitamin E, but not liproxstatin-1, in this context raises questions. The underlying mechanism may stem from liproxstatin-1’s specificity as a ferroptosis inhibitor, achieved through the trapping and stabilization of free radicals, which are not essential in this scenario. In contrast, vitamin E exhibits multifunctionality, including anti-inflammatory properties, interference with phospholipase A2 and ALOX, modulation of protein CD36 and low-density lipoprotein (LDLR) receptor expression, and regulation of protein kinase C (PRKC) signaling [216]. Specific preclinical studies have indicated that the administration of ferroptosis inhibitors or antioxidants can ameliorate organ damage and enhance survival in septic animal models [217–219]. This suggests that ferroptosis occurring within tissues, rather than within myeloid cells, may play a more significant role in driving the development of sepsis.

Ferroptosis appears to play a critical role in experimental non-septic multiorgan dysfunction rather than sepsis-induced multiorgan dysfunction. Highly soluble ferrostatin-analogue effectively blocks lipid peroxidation induced by the former and protects mice from injury and death, highlighting the importance of combinatorial treatment for sepsis [220]. Additionally, the synergistic role of different RCD pathways, such as the simultaneous neutralization of inflammatory factors, such as interleukin (IL)-1 and IL18, may further elucidate the pathophysiology of sepsis [221].

In conclusion, the dysregulation and interplay of ferroptosis and other forms of cell death contribute to tissue damage, organ dysfunction, and mortality in sepsis. Understanding the underlying mechanisms holds promise for the development of novel therapeutic strategies to improve outcomes in septic patients.

### Ischemia–reperfusion injury

Ischemia/reperfusion (I/R) injury occurs when blood supply to a tissue or organ is temporarily interrupted (ischemia) and then restored (reperfusion), leading to tissue damage and dysfunction. This phenomenon commonly occurs during medical conditions such as myocardial infarction, stroke, organ transplantation, and surgeries involving temporary occlusion of blood vessels [222].

Although the detailed mechanisms remain unclear, free radicals and calcium ion (Ca^2+^) loading are the main inducers of cell death, including ferroptosis, in myocardial I/R [223, 224]. In an ex vivo model simulating cardiac I/R injury in mice, iron chelators and inhibition of glutaminolysis attenuated cardiac injury induced by I/R [225]. Subsequent in vivo experiments in mice demonstrated that either iron chelation or pharmacologic blockade significantly alleviated chemotherapy- and IR-induced cardiomyopathy [226].

Kidney injury is another organ strongly associated with ferroptosis. Conditional knockdown of *Gpx4* in kidney tubular cells in mice induced lipid oxidation-dependent acute renal injury, whereas the use of liproxstatin-1 attenuated I/R-induced hepatic injury and cellular ferroptosis in *Gpx4* knockout mice [45]. Similarly, ferroptosis predominates in renal tubular cell death in severe I/R injury and oxalate crystal-induced acute renal injury mouse models, and the use of a ferroptosis-specific inhibitor (SRS16-86) has a potent protective effect against acute renal injury [227]. Acute renal injury is characterized by rapid onset and is accompanied by massive cell death and inflammatory responses, possibly due to the release of currently uncharacterized death signals from dead cells affecting the redox capacity of neighboring cells. A recent study has shown that platelet-activating factor (PAF) and PAF-like phospholipids mediate biofilm destabilization and propagate ferroptosis signals to neighboring cells. Conversely, this cascade can be inhibited by platelet-activating factor acetylhydrolase 2 (PAFAH2) or by antibodies targeting PAF, while knockdown or pharmacological inhibition of PAFAH2 enhances synchronized ferroptosis and exacerbates I/R-induced acute renal injury [228].

Ferroptosis also plays a critical role in cerebral [229], hepatic, and pulmonary I/R injures [125, 230]. The mitigation of I/R injury can be achieved by using lipophilic radical-trapping antioxidants such as liproxstatin to block the propagation of lipid peroxidation radicals or by inhibiting key enzymes, such as ACSL4, LPCAT3, ALOX5, and POR [231]. However, it is important to optimize the stability and specificity of antioxidant drugs in vivo and develop drugs that can exert anti-ferroptosis effects rapidly, as IR injury usually occurs within a short timeframe.

Overall, ferroptosis is increasingly recognized as a contributor to I/R injury in various tissues or organs, highlighting that inhibiting ferroptosis is a promising approach to prevent against I/R. Nevertheless, further studies are needed to elucidate how different forms of cell death, including ferroptosis and non-ferroptotic cell death, orchestrate the sterile inflammatory response, which is a central event in I/R. It is essential to identify specific serum or tissue biomarkers to distinguish between these types of cell death.

### Autoimmune disease

Autoimmune diseases encompass a spectrum of disorders arising from aberrant induction of cell death and inadequate clearance of self-cells or tissues, including systemic lupus erythematosus, rheumatoid arthritis, and multiple sclerosis [232]. Ferroptosis activation is evident in these autoimmune disease models [233]. Systemic lupus erythematosus, often referred to as lupus, is a chronic autoimmune disease that can affect various parts of the body, including the skin, joints, kidneys, heart, lungs, brain, and blood cells. In lupus, the body’s immune system mistakenly attacks its own tissues and organs, leading to inflammation and damage. Neutrophils from systemic lupus erythematosus patients exhibit heightened susceptibility to ferroptosis due to suppressed expression of GPX4 compared to healthy individuals. Serum autoantibodies and interferon-alpha enhance the binding of the transcriptional repressor CAMP responsive element modulator (CREM) to the *Gpx4* promoter, thereby inducing neutrophil ferroptosis. Moreover, specific knockdown of *Gpx4* in mouse neutrophils elicits clinical features resembling human systemic lupus erythematosus. Similarly, in progressive rheumatoid arthritis, synovial fibroblasts demonstrate abnormal proliferation dependent on the accumulation of ROS and lipid oxidation [234]. In the collagen-induced arthritis mouse model, imidazole ketone erastin (IKE) reduces the number of fibroblasts in the synovial membrane and alleviates arthritis progression [234].

Beyond synovial fibroblasts, other subpopulations and cellular heterogeneity are pivotal in controlling rheumatoid arthritis progression. For example, expression of transient receptor potential cation channel subfamily M member 7 (TRPM7) is elevated in articular chondrocytes from adjuvant arthritis rats, human rheumatoid arthritis patients, and erastin-treated cultured chondrocytes. Inhibition of TRPM7 activates the protein kinase C alpha (PRKCA)-NOX4 axis, leading to lipid ROS accumulation. Genetic or pharmacological inhibition of TRPM7 attenuates articular cartilage destruction in adjuvant arthritis rats [235]. Ferroptosis of anti-inflammatory M2 macrophages, but not M1 macrophages, positively correlates with rheumatoid arthritis disease severity. Treatment with liproxstatin-1 attenuates disease progression, accompanied by an increased proportion of M2 macrophages in K/BxN serum-transfer-induced arthritis mice [236].

These findings demonstrate the crucial role of ferroptosis activation in autoimmune diseases. Both rheumatoid arthritis and systemic lupus erythematosus patients display significantly elevated serum levels of ROS and lipid peroxidation. Therefore, targeting ferroptosis emerges as a promising therapeutic strategy to mitigate autoimmune diseases.

### Metabolic disorder

Metabolic disorders encompass disruptions in the body’s regulation of glucose, insulin, lipids, and other vital molecules. Common examples include metabolic dysfunction-associated steatotic liver disease (MASLD, also known as NAFLD) and diabetes mellitus (type 1 and type 2).

Contrary to ferroptosis’ role in eliminating cancer cells, its activation in normal liver cells can contribute to the development of liver diseases such as MASLD, a condition characterized by abnormal fat accumulation in the liver unrelated to alcohol consumption [237]. Non-alcoholic steatohepatitis (NASH), an advanced form of MASLD, can progress to liver cancer. While metabolic alterations due to free fatty acids largely drive hepatocyte injury, factors driving the transition from simple fatty liver to NASH are multifaceted. Lipotoxicity, oxidative stress, organelle dysfunction, and inflammation all contribute to hepatocyte death and worsen NASH progression, with ferroptosis playing a significant role [238–241].

ACSL4 is upregulated in patients with MASLD. Liver-specific deletion of *Acsl4* or pharmacological targeting with abemaciclib can significantly reduce steatosis and liver fibrosis in mice [242]. Additionally, interventions, such as rosiglitazone or genetic targeting of ACSL4, mitigate NASH and ferroptosis in animal models. Markers of oxidative stress, such as malondialdehyde and 4HNE, are elevated in NASH patients, indicating lipid peroxidation involvement [243]. Fluorescent probes tracking lipid droplets highlight ferroptosis’s pivotal role in MASLD [244, 245]. Antioxidants, such as vitamin E, reduce serum alanine aminotransferase (ALT) levels in NASH patients [246]. Stress responses accompany hepatocyte injury, as seen in the ATF4-dependent SLC7A11 expression in a NASH-related hepatocellular carcinoma mouse model [203]. In contrast to the accelerated liver injury observed in MASLD, a study reported progressive suppression of ferroptosis with the exacerbation of NASH [247]. This suggests a potential protective role of ferroptosis against liver injury in early-stage NASH. Further understanding the pathological role of ferroptotic damage in MAFLD may establish new approaches to prevent hepatocellular carcinoma.

The relationship between ferroptosis and diabetes is also well documented. First, excessive iron stores, including the expression of ferritin and ferroptosis-related genes, are positively associated with the development of type 2 diabetes mellitus in both mouse models and humans [248, 249]. Second, ferroptosis inducers, such as erastin, can impede the growth and function of human pancreatic islet-like cell clusters, which are three-dimensional structures resembling pancreatic islets in morphology and function [250]. Third, environmental pollutants such as acrolein and arsenic, which pose a high risk for type 2 diabetes mellitus, can induce ferroptosis and disrupt insulin secretion via the ER stress-related eukaryotic translation initiation factor 2 alpha kinase 3 (EIF2AK3; also known as PERK) pathway in mouse pancreatic β-cell MIN6 cells [251]. Moreover, ferroptosis inhibitors (e.g., ferrostatin-1) and antioxidants (e.g., quercetin) have shown protective effects against diabetic organ damage, including damage to the kidney, heart, liver, and brain [252–256].

These findings suggest the implication of ferroptosis and its associated proteins in metabolic disorders impacting glucose and lipid metabolism. Ferroptosis activation may initially suppress metabolic disorder progression but accelerate it in later stages. The main goal of studying ferroptosis in metabolic diseases is to understand the dynamic molecular mechanisms involved in disease development. This understanding can pave the way for the development of novel therapeutic interventions targeting ferroptosis, ultimately aimed at enhancing patient outcomes.

## Ferroptosis in cancer therapy

**Fig. 4:**
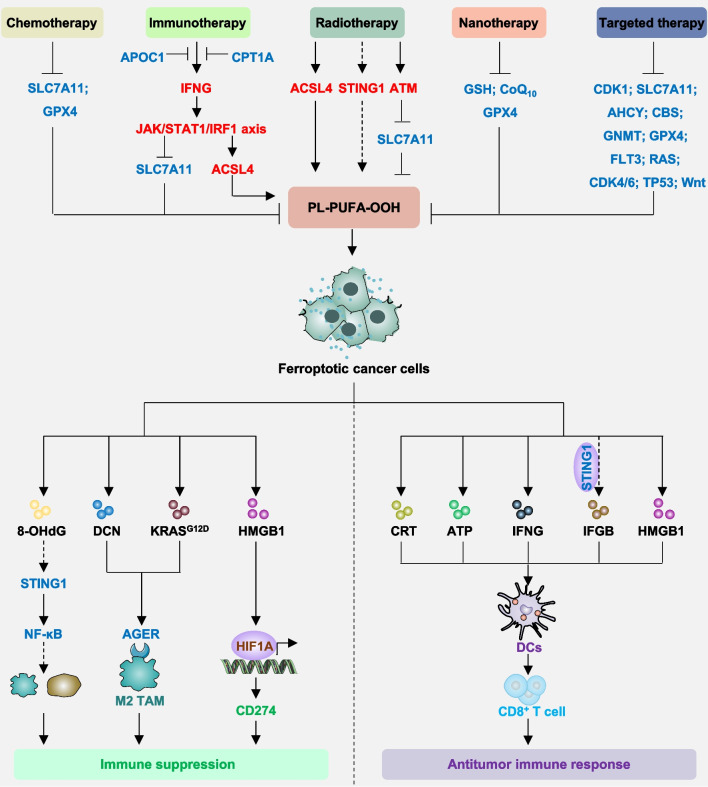
Ferroptosis in cancer therapy. Conventional cancer treatments, such as chemotherapy, radiotherapy, immunotherapy and nanotherapy can trigger ferroptosis, halting tumor growth. However, they may also activate pathways enabling cancer cells to evade ferroptosis. Therefore, combining therapies with the inhibition of ferroptosis escape pathways can significantly improve treatment outcomes. Ferroptotic cancer cells release damage-associated molecular patterns (DAMPs), which play a dual role in either promoting or inhibiting antitumor immunity, depending on the specific type and stage of cancer

Ferroptosis has attracted considerable attention in the field of cancer therapy due to its significant therapeutic potential. Currently, ferroptosis exhibits cancer-suppressive effects across various conventional cancer treatment modalities. As elucidated below, ferroptosis presents itself as a promising therapeutic avenue within specific cancer contexts (Fig. 4).

### Chemotherapy

Although chemotherapy remains the cornerstone of cancer therapy, it is often accompanied by significant side effects and the development of acquired drug resistance. Numerous preclinical and clinical investigations have been conducted to address drug resistance issues [257]. Ferroptosis has emerged as a promising strategy to address the challenges associated with current chemotherapy approaches, leveraging several key attributes: (1) selective targeting of malignant cells while preserving normal cellular integrity [258]; (2) reversal of drug resistance through ferroptosis induction alone [259], and (3) efficacy of certain Food and Drug Administration (FDA)-approved drugs in triggering ferroptosis [9]. For instance, ironomycin, a synthetic derivative of salinomycin, exhibits potent selective activity against mesenchymal stem cells from human breast cancer. Ironomycin accumulates and sequesters iron within lysosomes, subsequently activating ferritinophagy and blocking cancer cell proliferation [258]. Moreover, evidence suggests that several FDA-approved drugs can effectively induce ferroptosis in specific cancer types, offering cost-effective and side effect-reducing alternatives in drug development (Table 2).

Prolonged exposure to first-line chemotherapeutic agents often leads to multidrug resistance via various mechanisms, including increased autophagic activity. Given the dual role of autophagy, strategies targeting either autophagy inhibition or selective autophagy activation have shown promise in restoring sensitivity in drug-resistant cells. For instance, 4-octyl itaconate induces ferritinophagy-dependent ferroptosis, effectively overcoming carboplatin resistance in retinoblastoma cells and xenograft models [260, 261]. In macrophages, itaconate has been found to inhibit ferroptosis by activating the NFE2L2 pathway [262]. This suggests that itaconate could serve as an appealing agent for selectively inducing ferroptosis in cancer cells rather than immune cells, such as macrophages. Similarly, elevated phosphoglycerate dehydrogenase (PHGDH) levels contribute to resistance in castration-resistant prostate cancer patients against the androgen receptor inhibitor enzalutamide. Inhibition of PHGDH expression, either genetically or pharmacologically with NCT-503, has been shown to suppress CRPC cell growth and overcome Enza resistance in vitro and in vivo via ferroptosis activation [263]. Additionally, ferroptosis induction or NCT-503 treatment synergistically enhances enzalutamide-resistant CRPC cell sensitivity to enzalutamide in xenograft models [263]. These findings indicate the efficacy of ferroptosis induction as a standalone approach in eliminating drug-resistant cancer cells, with combinatorial therapies further augmenting chemotherapy outcomes.

In summary, ferroptosis represents a promising avenue in cancer therapy. However, due to tumor heterogeneity and feedback mechanisms influencing ferroptosis [10], leveraging combinatorial strategies and addressing tumor heterogeneity may enhance therapeutic efficacy further.

### Radiotherapy

Radiotherapy stands as the cornerstone treatment for several solid tumors, such as lung, breast, esophageal, colorectal cancers, and glioblastoma. Traditionally, its mechanism revolves around inhibiting tumor cell proliferation and growth by inducing apoptosis, primarily through damaging the DNA structure of tumor cells [264, 265]. Radiotherapy disrupts biomolecules, including lipids, via the excessive generation of ROS from cell water radiolysis, suggesting involvement of ferroptosis in radiotherapy. For instance, radiotherapy triggers ACSL4-dependent ferroptosis in various cancer cell lines and xenografted tumor-bearing mice [266]. However, the adaptive upregulation of SLC7A11 and GPX4 hinders radiotherapy efficacy; hence, inactivating SLC7A11 or GPX4 with ferroptosis inducers yields radioresistant cancer cells and xenograft tumors [266].

Moreover, combining radiotherapy with immunotherapy enhances cellular susceptibility to ferroptosis, independent of typical DNA damage effects. Radiotherapy-activated ATM serine/threonine kinase (ATM) produces interferon-gamma (IFNG, also known as IFNγ) independently but synergistically inhibits SLC7A11 and induces ferroptosis. The combination of immunotherapy-activated CD8^+^ T cells significantly inhibits B16F10, ID8, HT-1080 cancer cells, and xenografts in tumor-bearing mice [267]. Treatment with the Poly (ADP-Ribose) polymerase (PARP) inhibitor niraparib enhances radiotherapy-induced ferroptosis and boosts antitumor immune responses through the DNA sensor cyclic GMP-AMP synthase (CGAS) signaling pathway in colorectal cancer cells (MC38, CT26, and HT29) [268]. Thess study suggests inducing ferroptosis to enhance immunotherapeutic combinations with radiotherapy for cancer treatment.

Some metal ions can impede the anticancer effects of radiotherapy-dependent ferroptosis. For instance, radiotherapy induces a decrease in COMM domain-containing 10 (COMMD10), leading to intracellular copper accumulation and radio-resistance in hepatocellular carcinoma. Mechanistically, radiotherapy-induced low COMMD10 expression inhibits ubiquitin degradation of HIF1A, inducing copper accumulation and promoting nuclear translocation of HIF1A to induce transcription of ceruloplasmin (CP) and SLC7A11, thus inhibiting ferroptosis in hepatocellular carcinoma cells and tumor-bearing mice [269]. Elevated CP further promotes HIF1A expression by reducing iron, establishing a positive feedback loop [269]. Given that copper can also induce cuproptosis under certain conditions [106], it would be intriguing to delve deeper into elucidating the interplay between cuproptosis and ferroptosis in the context of radiotherapy.

Furthermore, targeting regulators of ferroptosis, such as AIFM2 [270], glutathione synthetase (GSS), GPX4, FA complementation group D2 (FANCD2), and MAF BZIP transcription factor F (MAFF) can sensitize specific cancers to radiotherapy-dependent ferroptosis [271–275]. Additionally, radiotherapy induces radiation colitis in patients, affecting their quality of life. However, an orally administered Pickering emulsion stabilized with halloysite clay nanotubes can inhibit ferroptosis, alleviating radiocolitis in vitro and in vivo [276]. This suggests that radiotherapy-dependent ferroptosis plays a dual role in cancer treatment, emphasizing the need for subsequent rational regulation of specific oxidative stress to safeguard the anticancer effects of radiotherapy.

### Immunotherapy

Unlike traditional cancer treatments, such as chemotherapy or radiation therapy, which directly target cancer cells, immunotherapy functions by activating the body’s immune system to identify and combat cancer cells more efficiently. Over the past decade, the emergence of immunotherapy, especially immune checkpoint inhibitors, has significantly altered the paradigm of tumor treatment; however, only a minority of patients exhibit responsiveness to immunotherapy. Consequently, addressing this challenge necessitates additional strategies to augment immunotherapy efficacy.

Recent research suggests that enhancing ferroptosis responses could potentially amplify the effectiveness of immunotherapy. For instance, anti-CD274-mediated immunotherapy triggers CD8^+^ T cells to release IFNG, subsequently inhibiting SLC7A11 and inducing ferroptosis, thereby enhancing immunotherapy in vivo [267]. ACSL4-dependent tumor ferroptosis, induced by the combination of IFN-gamma and arachidonic acid, also triggers CD8^+^ T cell-dependent antitumor immunity [277]. Similarly, targeting GPX4 in human triple-negative breast cancer has been shown to enhance the efficacy of anti-PDCD1 therapy [278]. Mefloquine, a medication primarily utilized for preventing and treating malaria, improves the effectiveness of anti-PD-1 immunotherapy [279]. This enhancement occurs through the IFNG-signal transducer and activator of transcription 1 (STAT1)-interferon regulatory factor 1 (IRF1)-LPCAT3-induced ferroptosis pathway in mouse models of melanoma and lung cancer [279]. Furthermore, inhibiting or remodeling immunosuppressive cells within the tumor microenvironment via ferroptosis can similarly augment immunotherapeutic effectiveness. For example, inhibition of apolipoprotein C1 (APOC1) restores ferroptosis sensitivity, leading to the conversion of tumor-associated macrophages from an M2 to an M1 phenotype and enhancing anti-PDCD1 immunotherapy against hepatocellular carcinoma and lung cancers [280, 281]. Inhibiting PGE2-induced ferroptosis in CD8^+^ T cells restores the anticancer activity of IL2 in mouse models [51]. Carnitine palmitoyltransferase 1A (CPT1A) is a key enzyme in fatty acid metabolism, facilitating the transfer of long-chain fatty acids into mitochondria for energy production via beta-oxidation. Its expression is regulated by MYC, which can activate the NFE2L2 pathway to inhibit ferroptosis [282]. In mouse models of lung cancer, targeting CPT1A with etomoxir induces ferroptosis and synergizes with anti-PDCD1 immunotherapy to enhance anti-tumor immunity [282]. These findings provide insights into a potential therapeutic strategy targeting fatty acid metabolism to enhance ferroptosis-mediated immune response.

Chimeric antigen receptor (CAR) T-cell therapy represents an innovative approach to immunotherapy, leveraging the body’s immune system against cancer. In this method, T cells, a subset of immune cells, are isolated from the patient’s bloodstream and genetically engineered to express CARs on their surface. These CARs empower the T cells to recognize and attack cancer cells bearing specific antigens. A recent study has presented initial evidence indicating that ferroptosis may curtail the persistence and anti-tumor efficacy of CAR-T cells. Conversely, inhibition of ferroptosis appears to foster the development of HNF1 homeobox A (HNF1A; also known as TCF1)^+^ hepatitis A virus cellular receptor 2 (HAVCR2; also known as TIM3)^−^ CAR-T cells, enhancing the therapeutic potential of CAR-T cell therapy [283]. Hence, inhibiting ferroptosis may augment CAR-T therapy.

In addition, the release of DAMPs from ferroptotic cells to promote immunogenic cell death (ICD) similarly enhances immunotherapy. For instance, in immunocompetent mice, early ferroptotic cells (1 h) but not late ferroptotic cells (24 h) promoted phenotypic maturation of bone marrow-derived dendritic cells (BMDCs) and induced a vaccination-like effect through the release of adenosine triphosphate (ATP) and HMGB1. Recombination-activated 2 (*Rag-2*) knockout mice failed to induce immunogenicity, suggesting that the mechanism of immunogenicity is tightly regulated by the adaptive immune system and is time-dependent [284]. Ferroptosis also impairs anti-tumor immunity by releasing DAMPs in a specific context. For instance, the release of KRAS^G12D^ protein from ferroptotic pancreatic cancer cells for uptake by macrophages via the AGER pathway leads to macrophage M2 polarization and stimulates tumor growth [202].

In addition to its role in inhibiting sorafenib-induced ferroptosis in hepatocellular carcinoma cells, metallothionein 1G (MT1G) is associated with ferroptosis response and acts as a prognostic biomarker and an indicator of immune checkpoint inhibitor therapy sensitivity in patients with prostate cancer [285]. In patients with triple-negative breast cancer, higher GPX4 expression is associated with lower cytolytic scores and poorer prognoses in immunotherapy cohorts [278]. This indicates GPX4’s role not just as a regulator, but also as a biomarker for predicting the immune response related to ferroptosis [278]. However, since GPX4 is widely expressed across both cancerous and immune cells, indiscriminate use of GPX4 inhibitors, such as RSL3, could lead to immunosuppression or toxicity [99, 286]. A recent study has shown that the compound N6F11 activates tripartite motif containing 25 (TRIM25), which predominantly exists in cancer cells rather than immune cells [287]. The activation of TRIM25 leads to the degradation of GPX4 via the ubiquitin–proteasome system (UPS) pathway [287]. Significantly, the combination of N6F11 with an anti-CD274 antibody has demonstrated potent anti-cancer efficacy in multiple pancreatic cancer mouse models, without discernible side effects [287].

In conclusion, ferroptosis has a dual role in immunotherapy [288], and the development of a specific inhibitor or inducer targeting ferroptosis can improve immunotherapy.

### Nanotherapy

Tumors and normal tissues possess distinct properties that can be exploited to design targeted delivery systems for improved therapeutic outcomes. Numerous studies have demonstrated the potential of nanomaterials targeting ferroptosis in the development of novel cancer therapies (Table 1). However, the design of such nanomaterials varies and is based on several key properties, including: (1) enhancing GSH depletion [289], (2) targeting iron metabolism and availability [290], and (3) targeting antioxidant defense and lipid peroxidation [291]. For instance, a Cu-tetra(4-carboxyphenyl) porphyrin chloride (Fe(III)) (Cu-TCPP(Fe)) metal–organic framework (MOF)-based nanosystem, incorporating gold nanoparticles (NPs) and RSL3, inhibits the tumor’s anti-ferroptosis pathway, thereby amplifying ferroptosis injury. Gold nanoparticles disrupt the pentose phosphate pathway, hinder GSH biosynthesis, and impede the cycling of CoQ_10_ to CoQ_10_H_2_. Meanwhile, copper oxidizes GSH to oxidized GSH (GSSG) and interacts with the GPX4 inactivation function of RSL3, significantly inducing ferroptosis in triple-negative breast cancer [292]. A novel drug delivery system named GDMCN2 gradually releases the sonosensitizer sinoporphyrin sodium and gemcitabine upon entry into tumor cells under ultrasound irradiation, leading to concomitant ROS generation. Mechanistically, ROS induce NCOA4-dependent ferritinophagy and exacerbate mitochondrial and DNA damage, thereby increasing the sensitivity of human pancreatic cancer cells and tumor-bearing mice to chemotherapeutic agents [293].

**Table 1:** Examples of nanomaterials for inducing ferroptosis

Nanoparticle	Encapsulation	Mechanism	References
Cisplatin-loaded iron oxide	Iron oxide	Fe^3+^ and ROS overload	[294]
Ferumoxytol	Fe_2_O_3_	Fe^3+^ and ROS overload	[295]
RSL3@COF-Fc	RSL3	Fenton reaction, GPX4 inhibition, and lipid peroxidation	[296]
DS@MA-LS	Doxorubicin and Sorafenib	ROS overload and SLC7A11 inhibition	[297]
FaPEG-MnMSN	Sorafenib	Fenton reaction and SLC7A11 inhibition	[298]
UCNP@LP(Azo-CA4)	Azobenzene combretastatin A4	Fenton reaction	[299]
BNP@R	RSL3	IFNG release and GPX4 inhibition	[300]
MnO_2_@HMCu_2_-xS	Mn^2+^, rapamycin	GSH depletion and ROS overload	[301]
Erastin@FA-exo	Erastin	GSH depletion and ROS overload	[302]
HA-C60-Tf/AS	Artesunate	Iron accumulation and GPX4 inhibition	[303]

Furthermore, synergies with other conventional treatments need to be considered alongside targeted strategies that directly trigger ferroptosis. For instance, a tumor microenvironment-degradable nanohybrid with dual radiosensitization modality overcomes enhanced ferroptosis defenses during radiotherapy in triple-negative breast cancer. Encapsulated l-butylsulfoximide (BSO) inhibits GSH biosynthesis and inactivates GPX4, while delivered ferrous ions act as potent ferroptosis response actuators via the Fenton reaction, significantly inducing a robust ferroptosis response. This synergistically interacts with surface-decorated gold (Au) nanoparticle-mediated radiosensitization, enhancing in vitro and ex vivo radiotherapy effects [304].

Overall, the field of nanotherapy, aimed at inducing ferroptosis to augment the efficacy of chemotherapy, radiotherapy, and immunotherapy, is rapidly advancing [305–311]. Nanotherapy presents multiple benefits, such as enhanced drug potency, minimized adverse effects, improved bioavailability, and the capacity to bypass biological obstacles such as the blood–brain barrier. Nonetheless, to mitigate off-target effects, the utilization of nanomedicine in human healthcare necessitates meticulous evaluation. Furthermore, the engineering of nanomaterials requires further refinement to ensure their appropriateness for clinical applications.

### Targeted therapy

Targeted therapy, a cancer treatment modality that employs drugs or other substances for the precise identification and eradication of cancer cells while sparing normal cells, contrasts traditional chemotherapy by specifically targeting molecular markers associated with cancer. These markers encompass proteins, genes, or elements of the tumor microenvironment that facilitate cancer proliferation and survival. Beyond previously discussed immune checkpoint inhibitors, ferroptosis plays a pivotal role in the realm of targeted therapy.

For instance, resistance to oxaliplatin in advanced colorectal cancer cases has been attributed to elevated expression of cyclin-dependent kinase 1 (CDK1), spurred by deletions in N6-methyladenosine modification. Both genetic and pharmacological interventions targeting CDK1 have been shown to re-establish oxaliplatin sensitivity in colorectal cancer cells [312]. Mechanistically, CDK1 interacts directly with ACSL4, phosphorylating it at Serine 447. This event recruits the E3 ubiquitin ligase ubiquitin protein ligase E3 component N-recognin 5 (UBR5), leading to the polyubiquitination of ACSL4 at lysines 388, 498, and 690, culminating in ACSL4 degradation. This degradation impedes lipid peroxidation and consequently ferroptosis [312]. It is noteworthy that while various inducers can activate ferroptosis, their specificities and direct targeting capabilities vary, as do the intrinsic properties of the tumor determining the drugs’ effectiveness in eliciting a ferroptotic response. For example, the overexpression of SLC7A11 in certain cancers can be targeted by sulfasalazine to induce ferroptosis [313], whereas lapatinib, despite its ferroptotic induction capability, does not directly target SLC7A11, potentially limiting its effectiveness [314]. In addition, targeting key enzymes in the transsulfuration pathway, such as adenosylhomocysteinase (AHCY; also known as SAHH), cystathionine β-synthase (CBS), and glycine N-methyltransferase (GNMT), can resensitize tumors to ferroptosis inducers targeting SLC7A11 [315–317]. In addition, sorafenib, a frontline treatment for advanced hepatocellular carcinoma, has demonstrated ferroptosis induction capabilities in liver, kidney [318] and pancreas by inhibiting system xc^−^ activity [319]. However, the efficacy of sorafenib is limited by acquired drug resistance associated with MT1G expression. Inhibition of MT1G expression enhances sorafenib’s anticancer activity through ferroptosis induction in vitro and in vivo [320]. In a pilot study involving patients with hepatocellular carcinoma treated with sorafenib, MT1G has been identified as a biomarker for predicting the development of resistance to sorafenib treatment [321].

Another noteworthy instance of targeted therapy associated with ferroptosis is imetelstat, a pioneering telomerase inhibitor that has demonstrated effectiveness across various blood cancer types. A recent study has elucidated imetelstat-induced ferroptosis mediated by ACSL4 as a mechanism of action specifically in acute myeloid leukemia [322]. However, the broader applicability of this ferroptotic effect to other telomerase inhibitors remains uncertain and warrants further investigation. Furthermore, imetelstat has been observed to induce apoptosis in leukemia stem cells within pediatric acute myeloid leukemia patient-derived xenografts [323], underscoring its potential as a multifaceted therapeutic agent in acute myeloid leukemia.

Synthetic lethality is defined within the molecular biology domain as a phenomenon where the simultaneous inactivation of two genes results in cell death, whereas inactivation of each gene individually does not significantly impact cell viability. This concept has been leveraged in the context of ferroptosis induction as a therapeutic strategy against specific cancer types. GPX4 inhibitors were identified through synthetic lethal screens aimed at finding compounds selectively toxic to cells harboring *RAS* mutations, illustrating the link between synthetic lethality and ferroptosis [324]. Recent studies have expanded the scope of synthetic lethality approaches to include the induction or enhancement of ferroptosis sensitivity. For instance, screenings have shown that simultaneous targeting of bromodomain and extra-terminal domain (BET) proteins and the proteasome markedly induces ferroptotic cell death in triple-negative breast cancer cells [325]. Furthermore, PARP inhibitors have been shown to induce ferroptosis by downregulating SLC7A11, acting synergistically with ferroptosis inducers in BRCA DNA repair associated (*BRCA*)-proficient ovarian cancer models [326]. In contrast, a recent study revealed that *BRCA1*-deficient breast cancer models display resistance to erastin-induced ferroptosis but exhibit vulnerability to GPX4 inhibitor-induced ferroptosis [327]. Targeting GPX4-mediated ferroptosis defense mechanisms could potentially enhance the sensitivity of PARP inhibitors [327]. Simultaneously targeting CDK4/6 and BRD4 enhances the susceptibility of pancreatic and breast cancer cells to senescence and ferroptosis [328]. The dual-targeting PI3K and histone deacetylase (HDAC) inhibitor, BEBT-908, triggers ferroptosis and boosts antigen presentation, thereby enhancing the antitumor immune response in experimental cancer models [329]. The transcription factor CCAAT-enhancer binding protein alpha (CEBPA) plays a crucial role in both normal and leukemic cell differentiation. Moreover, CEBPA-dependent expression of Fms related receptor tyrosine kinase 3 (FLT3) renders acute myeloid leukemia cells vulnerable to ferroptosis upon inhibition of FLT3 [330], suggesting a therapeutic potential for targeting this pathway in acute myeloid leukemia treatment. Furthermore, research has identified mutations in various oncogenes and tumor suppressor genes, such as *RAS* and *TP53* [9, 331–334], aberrant embryonic developmental signals, including the Wnt/β-catenin and Hippo signaling pathways [334–336], and specific gene variants such as the androgen receptor [337], as factors that influence the sensitivity of tumor cells to ferroptosis. This insight provides opportunities to develop targeted strategies that enhance the effectiveness of therapies reliant on ferroptosis.

In summary, the role of ferroptosis in targeted therapy represents a promising frontier in cancer treatment, offering a novel mechanism to kill cancer cells through iron-dependent lipid peroxidation. Ongoing research into ferroptosis mechanisms, susceptibility factors, and potential inhibitors or inducers continues to enrich our understanding and application of this process in the context of precision oncology.

## Ferroptosis and ICD

**Fig. 5:**
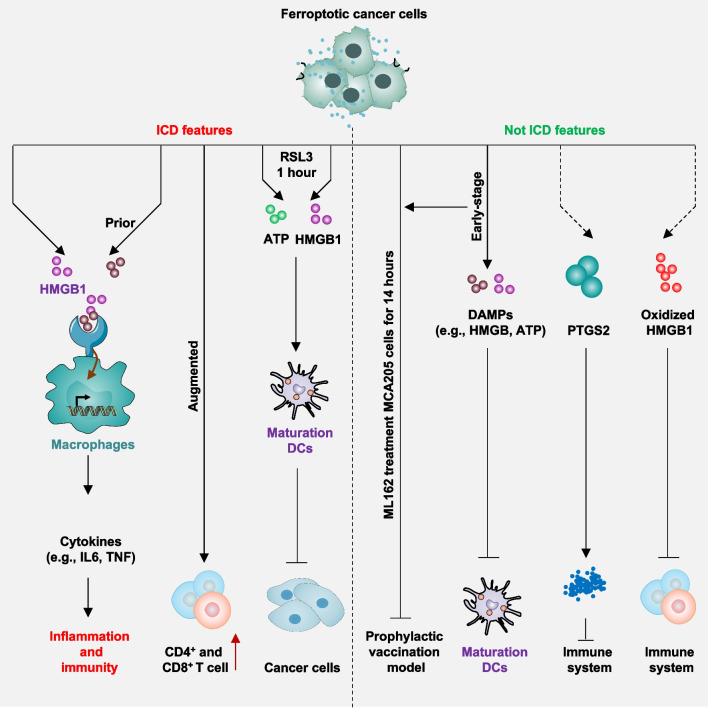
Relationship between ferroptosis and immunogenic cell death. Ferroptotic cancer cells release damage-associated molecular patterns (DAMPs), expose calreticulin (CRT), and demonstrate some level of immunogenicity, resembling a form of immunogenic cell death (ICD). However, the challenges encountered in prophylactic vaccine trials and the observed immunosuppressive effects associated with increased PTGS2 and PGE2 expression, oxidized HMGB1, and phospholipid peroxidation during ferroptosis suggest that ferroptosis may not fully meet the criteria for being classified as ICD

ICD is a form of cell demise that triggers the adaptive immune response against cancer cells. In contrast to some cell death modalities that occur stealthily without arousing immune surveillance, ICD entails the discharge of specific molecules termed DAMPs from the dying cells. DAMPs function as signaling molecules, alerting the immune system and autophagy to the presence of cellular damage or stress [338]. Although ferroptotic cancer cells release certain DAMPs, the classification of ferroptosis as a form of ICD remains contentious (Fig. 5). This section will delve into the debated perspective concerning ferroptosis’s association with ICD.

### Supportive views

ICD is characterized by three hallmark features: (i) the emission of ‘find me’ signals, which recruit immune cells such as dendritic cells and macrophages to the damage site, catalyzing the release of inflammatory mediators and triggering an adaptive immune response; (ii) the exposure of ‘eat me’ signals on the cell surface, enhancing the immunogenic profile of the cell; and (iii) the secretion of ‘danger alert’ signals through the release of DAMPs [339]. When ferroptotic cancer cells fulfill these criteria, they qualify as instigators of ICD.

DAMPs are endogenous molecules that can initiate and perpetuate a non-infectious inflammatory response in the absence of pathogenic infection [340]. DAMPs are released by stressed, damaged, or dying cells and can be recognized by pattern recognition receptors on cells of the innate immune system, such as dendritic cells, macrophages, and neutrophils. In the context of tumor immunity, DAMPs, play a pivotal role in activating, differentiating, and maturing antigen-presenting cells [341]. Key DAMPs include HMGB1, ATP, heat shock proteins (HSP70 and HSP90), and DNA. Ferroptotic damage in HT-1080 and PANC-1 cells has been demonstrated to promote the release of HMGB1 through autophagy, which in turn drives macrophage-mediated inflammatory responses via the AGER receptor [342]. Similarly, ferroptotic cancer cells, including HT-1080, PANC-1, HeLa, and KPC lines, have been observed to release the proteoglycan decorin (DCN), which interacts with the AGER receptor on macrophages [343]. This interaction initiates the production of pro-inflammatory cytokines and amplifies antitumor immunity through the activation of the nuclear factor kappa B (NF-κB) signaling pathway [343]. Intriguingly, the precedence of DCN release over HMGB1 suggests its potential as an early disease detection marker [343]. The administration of RSL3 in head and neck squamous cell carcinoma xenograft models not only diminishes suppressive immune cell populations within the tumor microenvironment but also increases the numbers of CD4^+^ and CD8^+^ T cells. [344]. Furthermore, treatment with RSL3 for 1 h induced a notable release of HMGB1 and ATP from ferroptotic MCA205 and GL261 cells, which facilitated the maturation of dendritic cells and suppressed tumor growth in a vaccine tumor mouse model [284, 345]. Combined with the evidence from non-tumor models demonstrating that ferroptotic damage leads to sterile inflammation and immune cell infiltration in various types of tissue damage, ferroptosis is thereby identified as a form of ICD.

### Non-supportive views

To elucidate the role of ferroptosis in initiating ICD, a study employing an inducible ferroptosis model identified three distinct phases: an ‘initial’ phase marked by lipid peroxidation, an ‘intermediate’ phase characterized by ATP release, and a ‘terminal’ phase defined by HMGB1 release and loss of plasma membrane integrity [346]. These findings indicate that ferroptosis can circumvent cancer cell resistance to death; however, its immunogenic potential in a prophylactic vaccination model, specifically using ML162-treated MCA205 cells for 14 h, appeared limited [346]. This observation contrasts with previous findings utilizing RSL3-treated MCA205 cells for 1 h [284]. Additionally, while early-stage ferroptotic cancer cells do release DAMPs, such release seems to inhibit dendritic cell maturation and function. This inhibition could result from incomplete ferroptosis in cancer cells briefly treated with RSL3 and then injected into mice, inducing an unclear response [346]. Another consideration is the dual role of HMGB1 in tumor immunity, which varies with its redox state: reduced HMGB1 stimulates the immune system, whereas oxidized HMGB1 may suppress it [347]. The conventional enzyme-linked immunosorbent assay (ELISA) method for HMGB1 detection does not differentiate between these redox states, raising questions about the potential shift from a reduced to an oxidized form over extended stimulation periods.

The immunogenic effect of DAMPs could be counteracted by the expression of certain molecules during ferroptosis, such as PTGS2, which facilitates PGE2 production and consequently suppresses immune responses by inducing ferroptosis in CD8^+^ T cells [51, 348, 349]. The DAMPs’ release from ferroptotic cells, following plasma membrane rupture due to lipid peroxidation, along with possible oxidative byproducts or unknown immunosuppressive DAMPs, may further hinder immune cell maturation and function [63, 350, 351].

The ongoing scientific discourse regarding whether ferroptotic death elicits immunostimulation or immunosuppression persists. These variances may stem from discrepancies in experimental models and the diverse oxidative environments influencing the activity of DAMPs.

## Pharmacological modulation of ferroptosis

Pharmacological modulation of ferroptosis presents a promising therapeutic strategy for various diseases associated with dysregulated ferroptosis. Numerous clinical drugs and biologically active pharmacological agents have been identified as modulators, both inducers (Table 2) and inhibitors (Table 3), of ferroptosis. In this context, we provide a summary of classical modulators targeting the core pathway of ferroptosis.

**Table 2:** Examples of ferroptosis inducers

Inducers	Target	Function	References
Sulfasalazine	SLC7A11	Inhibit cystine uptake	[183]
Cisplatin	GSH	Induce lipid peroxidation	[352]
Dihydroartemisinin, artesunnate	Ferritin	Increase iron overload	[353]
Statins	HMGCR, GPX4	Block GPX4 production	[40]
Gefitinib	ROS	Induce lipid peroxidation	[354]
Zalcitabine	Mitochondrial DNA stress	Induce STING1-mediated autophagy	[355]
Sorafenib	SLC7A11	Inhibit cystine uptake	[320]
Lapatinib, siramesine	Fe	Increase iron overload	[356]
Haloperidol	Dopamine receptor D2	Induce ER stress-dependent autophagy	[357]
Paclitaxel	TP53, SLC7A11	Inhibit cystine uptake and induce iron overload	[358]
Altretamine	GPX4	Inhibit GPX4 activity	[359]
Brefeldin A	Golgi	Induce Golgi stress	[360]
Buthionine sulfoximine (BSO)	GCL	Inhibit GSH produciton	[183]
Doxorubicin	HMOX1	Increase iron overload	[226]
Salinomycin	Ferritin	Induce ferritinophagy	[258]
Erastin	System xc^−^	Inhibit cystine uptake	[9]
FIN56	GPX4	Induce GPX4 degradation and inhibit the mevalonate pathway	[40]
FINO2	Fe	Increase iron overload and induce GPX4 degradation	[361]
iFSP1	AIFM2	Inhibit AIFM2	[107]
ML162, ML210, RSL3, diacylfuroxans, BCP-T.A	GPX4	Inhibit GPX4 activity	[97, 362, 363]
dGPX4, DC2, 8e	GPX4	Induce PROTAC-dependent GPX4 degradation	[364–366]
N6F11	GPX4	Induce TRIM25-dependent GPX4 degradation	[287]
Imidazole ketone erastin (IKE)	SLC7A11	Inhibit cystine uptake	[367]
FSEN1	AIFM2	Inhibit AIFM2	[368]
Metformin	SLC7A11	Inhibit UFMylation of SLC7A11	[369]
Rapamycin	GPX4	Induce autophagy-dependent GPX4 degradation	[98]
Dimethyl fumarate	ALOX5, STAT3	Induce ALOX5-dependent lipid peroxidation	[370]
Itaconic acid	Ferritin	Induce ferritinophagy	[260]
BAY 87-2243	Mitochondrial complex I	Induce mitochondrial-dependent ROS elevation	[371]
MMRi62	Ferritin	Induce ferritinophagy	[372]
NC-R17	GPX4	Induce proteasomal degradation of GPX4 via PROTAC	[373]

**Table 3:** Examples of ferroptosis inhibitors

Class	Name	References
ACSL4 inhibitor	Triacsin C, troglitazone, rosiglitazone	[374–376]
ALOX inhibitor	Zileuton, MK886, PD146176, baicalein, LOXBlock-1	[33, 42, 45, 377, 378]
DPP4 inhibitor	Vildagliptin, alogliptin, linagliptin	[30, 360]
Iron chelator	Deferoxamine (DFO), 2,2-bipyridyl, ciclopirox olamine (CPX), deferiprone, pioglitazone	[9, 324, 379, 380]
Lipid peroxidation inhibitor	Ferrostatins (e.g., ferrostatin-1, SRS11-92, SRS12-45, SRS13-35, SRS13-37, and SRS16-86), liproxstatin-1	[9, 45]
Lysosome and autophagy inhibitor	Ammonium chloride, bafilomycin A1, chloroquine, wortmannin, 3-methyladenine, cryptotanshinone, CA-074Me, pepstatin A, E64	[381–385]
Mitochondrial ROS inhibitor	XJB-5–131, JP4-039, mitoTEMPO, MitoQ	[226, 386, 387]
NOX inhibitor	2-acetylphenothiazine, diphenyleneiodonium chloride (DPI)	[9, 30]
Broad-spectrum antioxidant	Vitamin E, vitamin K, n-acetylcysteine (NAC), butylated hydroxyltoluene (BHT), GSH and CoQ_10_	[9, 108, 388–390]
FDFT1 inhibitor	Zaragozic acid A, YM-53,601	[40]
STING1 inhibitor	H-151	[355]
CTSB inhibitor	CA-074Me	[181]

### Inducers

1. Inhibition of systems xc^−^. In the pursuit of a synthetic lethal agent targeting *KRAS*-mutated human foreskin fibroblasts (BJeLR) cells, the ferroptosis inducer erastin was initially reported in 2003 [391]. Erastin functions by depleting GSH primarily through inhibition of the system xc^−^, which serves as a cofactor in the synthesis of GPX4 [9]. Erastin exhibits poor metabolic stability and water solubility in vivo; however, its analogs such as piperazine erastin and IKE have been developed to mitigate these limitations [392]. Additionally, erastin can inhibit VDAC2 and VDAC3, thereby promoting the accumulation of endogenous ROS [388]. Several FDA-approved drugs, such as sorafenib [393], sulfasalazine [313], metformin [369], and acetaminophen [167], also induce ferroptosis in several cancer and non-cancer cells by inhibiting system xc^−^. However, it is noteworthy that one study suggested sorafenib may lack activity in inhibiting system xc^−^ to induce ferroptosis in certain cancer cells (e.g., HT-1080, A375, A549, HT29, MDA-MB-436, and Hep G2) [394]. Furthermore, sensitivity to ferroptosis is increased by inhibiting glutamate-cysteine ligase catalytic subunit (GCLC), the rate-limiting enzyme in GSH synthesis, using compounds such as buthionine sulfoximine (BSO). It’s worth noting that SLC7A11 inhibition-induced GSH depletion or lipid peroxidation under specific conditions doesn’t necessarily indicate the occurrence of ferroptosis. Moreover, genetic or pharmacological inhibition of SLC7A11 can trigger cell death that is not reversible by ferrostatin-1 [104, 395]. Possible explanations include the varying unknown off-target effects of different SLC7A11 inhibitors, which may induce non-ferroptotic cell death. Additionally, SLC7A11 plays a broad role in amino acid uptake, extending beyond cysteine alone. Moreover, cell density is another important factor influencing erastin’s effect [336].
2. Inhibition of GPX4. There are three approaches to inhibit the GPX4 pathway and induce ferroptosis. Covalent inhibitor: The first GPX4 inhibitors obtained through high-throughput screening include RSL3 and ML162 [97]. ML162 shares structural and biological properties with RSL3 but exhibits improved activity. These covalent inhibitors induce ferroptosis by binding to the selenocysteine residue Sec46, although they lack a drug-like pocket, leading to issues with low selectivity and poor pharmacokinetic properties that require optimization [396]. Recent studies using co-crystal structure assays combined with mutation assays in HT-1080 fibrosarcoma cells, suggest that the region around Cys66 also serves as a covalent binding site for RSL3 and ML162 on GPX4, indicating they have multiple binding sites on GPX4 [93]. Another covalent inhibitor operates within the intracellular system without direct binding ability to GPX4 in cell-free systems. These GPX4 inhibitors include nitrile oxide electrophilic inhibitors (e.g., ML210 [362] and diacylfuroxans [363]) and hetero-aromatic electrophilic inhibitors (e.g., BCP-T.A [397]), offering improved physicochemical and pharmacological properties. PROTAC degraders: Proteolysis-targeting chimeras (PROTACs) are small molecule compounds engineered to degrade specific target proteins within cells via the UPS. They consist of three components: a “warhead” binding to the protein of interest, an E3 ubiquitin ligase-recruiting ligand, and a chemical linker connecting them. Examples of GPX4-PROTAC degraders include dGPX4 [364], DC2 [365], and 8e [366], which utilize ML162, ML210, and RSL3 as warheads. Cell type-specific degraders: While covalent inhibitors and PROTAC degraders hold promise for inhibiting tumor growth, they may affect both cancerous and normal cells due to the widespread expression of GPX4. In contrast, as discussed above, N6F11 represents the first cell type-dependent ferroptosis inducer by selectively degrading GPX4 in cancer cells through the action of TRIM25, exhibiting efficacy and safety simultaneously [287].
3. Depletion of CoQ_10_. CIL56 and FIN56 induce ferroptosis potentially by activating rate-limiting enzymes of the cholesterol synthesis pathway, such as farnesyl-diphosphate farnesyltransferase 1 (FDFT1), and by depleting CoQ_10_ to promote GPX4 degradation; however, the precise mechanisms require further elucidation [40]. These observations indicate that fatty acid synthesis and the mevalonate pathway regulate susceptibility to ferroptosis through distinct mechanisms. Consequently, certain clinical anti-lipid drugs, such as statins, induce ferroptosis by inhibiting the rate-limiting enzyme of the mevalonate pathway, 3-hydroxy-3-methylglutaryl-CoA reductase (HMGCR), resulting in CoQ_10_ depletion [398]. Another key pathway parallel to GPX4 is the AIFM2-CoQ_10_ axis, and ferroptosis sensitizer 1 (FSEN1) can noncompetitively target AIFM2 to sensitize cancer cells to ferroptosis [368]. Nevertheless, the in vivo efficacy of AIFM2 inhibitors requires optimization. Conversely, icFSP1, a potent AIFM2 inhibitor, selectively induces ferroptosis via phase separation in *GPX4* knockdown Pfa1 and HT-1080 cells overexpressing AIFM2 [110].
4. Induction of iron overload and peroxides accumulation. Compounds that promote iron overload or induce lipid peroxidation play crucial roles as ferroptosis inducers. For instance, excessive nonheme iron accumulation can trigger ferroptosis in various cell types and tissues, including mouse cardiomyocytes [226], hepatocytes [399], bone marrow macrophages and organotypic hippocampal slice cultures [400, 401]. Additionally, FINO2, an organic peroxide featuring a 1,2-dioxolane skeleton, exerts a dual mechanism for inducing ferroptosis by directly promoting iron oxidation or indirectly inhibiting GPX4 activity [361]. Furthermore, artemisinin and its derivatives (such as dihydroartemisinin and artesunate) are capable of effectively inducing ferroptotic cell death in cancer cells [353].

### Inhibitors

1. Iron chelators. Iron chelators function by sequestering iron ions within cellular or tissue compartments, thereby diminishing their availability for a diverse array of biochemical pathways. Deferoxamine, deferiprone, and cyclipirox are esteemed examples of iron chelators recognized for their efficacy in mitigating ferroptosis [9, 402]. While iron chelators have shown promise in preclinical studies for ferroptosis inhibition, translating these findings into clinically viable therapies poses challenges. In addition, iron chelators may bind to other metal ions besides iron, leading to off-target effects and potential toxicity. Ensuring specificity for iron is crucial to minimize adverse effects.
2. Radical-trapping antioxidants. Radical-trapping antioxidants play a crucial role in capturing and stabilizing free radicals through electron donation or hydrogen atom transfer mechanisms. This process effectively converts free radicals into stable molecules, thereby interrupting the propagation of oxidative chain reactions. Notable examples of radical-trapping antioxidants known to inhibit ferroptosis include α-tocopherol (vitamin E), GSH, CoQ_10_, flavonoids, carotenoids (such as β-carotene), polyphenols (such as resveratrol), liproxstatin-1, and ferrostatin-1 [9, 33, 45]. Among these, liproxstatin-1 and ferrostatin-1 are widely utilized radical-trapping antioxidants for ferroptosis inhibition across various experimental models. Liproxstatin-1, specifically, has demonstrated potent anti-ferroptotic effects at nanomolar concentrations in intestinal, cardiac, and murine models of ischemia–reperfusion injury [374, 403]. Ferrostatin-1 functions by preventing lipid membrane damage through a reductive mechanism, albeit it exhibits metabolic instability and is predominantly used in in vitro experimental settings. Structural studies on ferrostatin-1 have highlighted the importance of primary aromatic amines in its ability to inhibit ferroptosis. Additionally, 7-dehydrocholesterol, a precursor of cholesterol, has emerged as an endogenous radical-trapping antioxidant capable of suppressing ferroptosis in various cancer cell lines and mitigating ischemia–reperfusion injury in kidneys. Similarly, two tryptophan metabolites, serotonin and 3-hydroxy-o-cyclohexanecarboxylic acid, function as radical-trapping antioxidants to mitigate lipid peroxidation, consequently inhibiting ferroptosis in numerous cancer cell lines [174]. The role of serotonin as an endogenous antioxidant in ferroptosis relies on solute carrier family 6 member 4 (SLC6A4) transport across the plasma membrane. This has been validated in K562 chronic myelogenous leukemia cells through CRISPR interference/activation screening, suggesting that lipid peroxides contributing to ferroptosis may be situated at intracellular membranes [404]. Furthermore, in addition to radical-trapping antioxidants, several natural or synthetic antioxidants, such as acetylcysteine (N-acetylcysteine) [49] and the mitochondrial antioxidant mito-TEMPO [405], demonstrate the capacity to inhibit ferroptosis under specific conditions. Nevertheless, there persists a necessity for systematic comparisons evaluating the efficacy of endogenous versus exogenous antioxidants against ferroptosis. The clarification is also needed on whether all forms of cell death inhibited by radical-trapping antioxidants correspond to instances of ferroptosis.
3. Enzyme inhibitors. ACSL4-mediated CoA linkage to PUFAs, such as arachidonic acid, adrenic acid, and eicosapentaenoic acid, serves as an initial step in the cascade that triggers ferroptosis, subsequently leading to the activation of membrane lipid peroxidation facilitated by enzymes including LPCAT3, POR, or ALOXs [35]. Consequently, inhibitors targeting ACSL4 (e.g., troglitazone, rosiglitazone, and triacsin C) or ALOXs (e.g., baicalein, PD146176, zileuton, AA-861, and ML351) demonstrate efficacy in suppressing ferroptosis. Noteworthy is the role of arachidonic acid as a substrate for ACSL4, which can feedback-enhance the ubiquitinated degradation of ACSL4, suggesting the involvement of non-ACSL4-dependent compensatory pathways in promoting ferroptosis [406, 407]. Conversely, SCD-dependent synthesis of MUFAs exerts an inhibitory effect on ferroptosis, hence inhibition of SCD, exemplified by A939572, enhances sensitivity to ferroptosis [53]. Furthermore, selenium acts synergistically with GPX4, increasing its abundance and augmenting the scavenging of lipid peroxidation [408].

## Biomarkers of ferroptosis

While certain distinctive biochemical characteristics, genetic alterations, and changes in cellular morphology differentiate ferroptosis from other forms of RCDs [9], effectively monitoring ongoing ferroptosis in vivo or identifying cells sensitive to ferroptosis holds significant promise. The accumulation of excessive lipid peroxides emerges as a fundamental event of ferroptosis. Specific markers of ferroptosis, such as oxidized PUFA-phospholipids or their derivatives, along with oxidation products such as 4HNE, 8-hydroxy-2′-deoxyguanosine (8-OHdG), and malondialdehyde, have been proposed [202, 367, 409]. However, the challenge lies in discerning the threshold at which ferroptosis occurs in vivo, given that these substances can also be generated under physiological conditions [410].

Furthermore, proteins such as extracellular DCN released by ferroptosis-prone PDAC cells and TFRC accumulation on the plasma membrane have been identified [343, 411]. Yet, limitations including timing of detection, cellular variances, predominant initiation mechanisms, and other factors may impede their utility (Table 4).

**Table 4:** Examples of ferroptosis biomarkers

Name	Property	Model	References
4HNE	Oxidation product	Broad-spectrum cell lines, in vivo model	[19, 409, 412, 413]
MDA	Oxidation product	Broad-spectrum cell lines, in vivo model	[53, 390, 414, 415]
8OHdG	Oxidation product	Broad-spectrum cell lines, in vivo model	[170, 416, 417]
DCN	Protein	HT-1080, PANC-1, Bx-PC3 and NCI-H460 cell lines, in vivo model	[343]
TFRC	Protein	HT-1080 cell line, in vivo model	[411]
PRDX3	Protein	HT-1080, A549, Huh7, HT29 and SV589 cell lines, mouse models of AFLD and MASLD	[418]

Recently, hyperoxidized peroxiredoxin 3 (PRDX3) has emerged as a ferroptosis-specific marker [418]. Hyperoxidized PRDX3 is observable in cells treated with ferroptosis inducers such as erastin, RSL3, or FIN56, distinguishing it from other RCDs, such as apoptosis or necroptosis [418]. Its use as a marker has shed light on ferroptosis as the causative mechanism of hepatocyte death in alcoholic and non-alcoholic fatty liver disease in mice. However, its prevalence in human tissue samples, particularly tumor samples, remains uncertain.

While GSH and GPX4 play roles in regulating various forms of cell death, they are commonly employed for ferroptosis monitoring. Traditional methods have relied on Western blotting for GPX4 protein expression analysis and kits for total GSH quantification, both of which require cell inactivation. However, accurately targeting cancer cells requires real-time monitoring of GPX4 and organelle-specific GSH levels in living cells. The application of the fluorescent probe QP1TF allows non-invasive, continuous assessment of GPX4 distribution and abundance in live HEK293, HT1080, CT26, HeLa, and SKOV3 cells. This approach aids in predicting the susceptibility of tumor cells to ferroptosis-inducing drugs targeting GPX4, such as altretamine and sorafenib [419]. Similarly, a study utilizing an in silico rhodamine GSH reversible probe, binding to Halo Tag proteins, demonstrated the activation of GSH probes specifically within the nucleus, influencing cell proliferation independently. This innovative method of organelle-specific detection holds promise for inspiring the development of similar detectors for various cellular components [420].

In summary, biomarkers serve as critical instruments in ferroptosis research, offering insights into disease mechanisms, prognosis, treatment response, and drug development. While discussed biomarkers hold potential for monitoring ferroptosis, comprehensive studies are warranted to discern lipid, genetic, and protein changes specific to ferroptosis in comparison to other forms of cell death.

## Crosstalk between ferroptosis and other RCD pathways

**Fig. 6:**
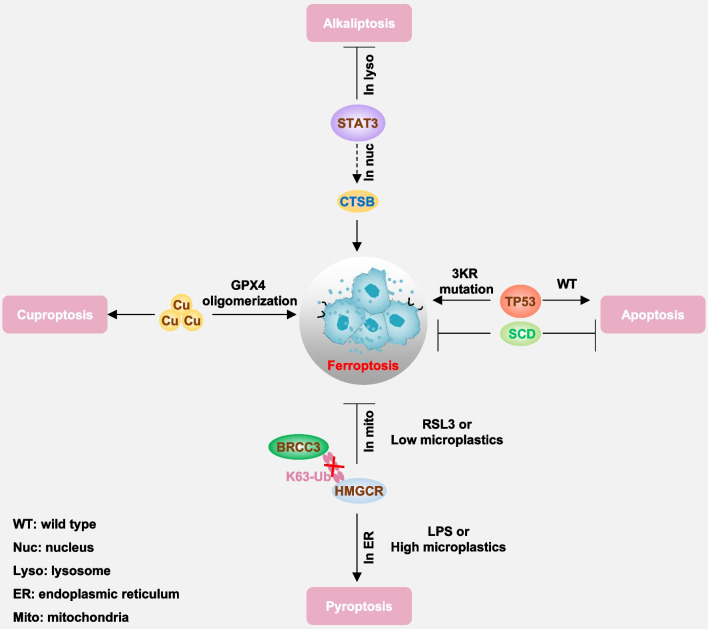
Crosstalk between ferroptosis and other RCD pathways. Many proteins or mental ions are multifunctional, tandem with ferroptosis and alkaliptosis, cuproptosis, pyroptosis and apoptosis

Ferroptosis also engages in crosstalk with other RCDs via specific molecular pathways (Fig. 6). For instance, the BRCA1/BRCA2-containing complex subunit 3 (BRCC3; also known as BRCC36) deubiquitinates HMGCR, a process dependent on deubiquitinating enzyme (DUB) activity, thereby inhibiting ferroptosis in human hepatocellular carcinoma cells while promoting cellular pyroptosis [421]. This antagonistic effect is contingent upon the concentration and type of inducers, regulating the organelle localization of HMGCR, suggesting either an off-target effect or involvement of other molecular players. Conversely, the release of DAMPs from ferroptotic cancer cells induces ICD and promotes cell demise. Additionally, ferroptosis and apoptosis intersect via shared regulatory factors and pathways. For example, *TP53*, a pivotal gene mediating autophagy and apoptosis [422], induces ferroptosis rather than apoptosis in response to oxidative stress, mediated by its acetylated mutant *TP53(3KR)*, which suppresses the expression of SLC7A11 in mouse embryonic fibroblasts and human U2OS cells [331]. Moreover, F-box and WD repeat domain containing 7 (FBW7) inhibits SCD expression through the suppression of nuclear receptor 4 subfamily A group 1 (NR4A1), concurrently regulating apoptosis and ferroptosis in human pancreatic cancer cells (PANC1 and SW1990) [423]. However, FBW7 may exhibit either an anti- or pro-ferroptotic effect depending on downstream substrates and cellular contexts [424]. Several members of the BCL2 family, crucial regulators of apoptosis sensitivity, are also implicated in regulating ferroptosis. For instance, in vitro studies have demonstrated that a Bruton’s tyrosine kinase inhibitor, zanubrutinib, synergistically enhances the anti-tumor effects of a BCL2 apoptosis regulator (BCL2) inhibitor, navitoclax [425]. This synergy is attributed to increased apoptosis and ferroptosis induced by preferential ROS generation, particularly effective in double-hit lymphoma [425]. Moreover, erastin-induced ferroptosis in neuronal cells was concomitant with BH3 interacting domain death agonist (BID) transactivation to mitochondria, resulting in mitochondrial membrane potential loss, augmented mitochondrial fragmentation, and diminished ATP levels [426]. These observations indicate the involvement of mitochondria in linking ferroptosis and apoptosis, with decreased mitochondrial membrane potential serving as a shared signal in these cell death pathways. However, distinguishing caspase-independent apoptosis from ferroptosis remains a challenge. In kidney damage models, the induction of ferroptosis and necroptosis is commonly observed [427, 428]. This suggests that specifically targeting these two pathways could be effective in preventing kidney damage.

Ferroptosis is also an autophagy-dependent form of cell death [429, 430], where increased autophagic flux promotes ferroptosis by selectively degrading antiferroptotic proteins or organelles, such as ferritin, SLC40A, and lipid droplets [77, 202, 367, 431–436]. In addition, lysosomal membrane permeabilization mediated by signal transducer and activator of transcription 3 (STAT3) induces CTSB expression, thus facilitating ferroptosis [181, 381]. CTSB and STAT3 also participate in regulating alkaliptosis in human pancreatic ductal adenocarcinoma cells, suggesting potential interaction between ferroptosis and alkaliptosis through STAT3 or pH-dependent mechanisms [437]. Cyclophosphamide, an alkylating agent used in chemotherapy, triggers the degradation of GPX4 in leukemia cells. This degradation ultimately leads to parthanatos through the activation of apoptosis inducing factor mitochondria associated 1 (AIFM1) [438]. Additionally, excess copper induces cuproptosis by promoting abnormal oligomerization of lipoylated proteins in the tricarboxylic acid cycle, along with reducing levels of Fe-S cluster proteins, with ferredoxin 1 (FDX1) playing a pivotal role in regulating cuproptosis [439]. Copper also triggers GPX4 ubiquitination and the formation of GPX4 aggregates, leading to autophagy-dependent ferroptosis [440, 441]. Conversely, sorafenib and erastin enhance cuproptosis in primary hepatocellular carcinoma by promoting copper-dependent aggregation of lipoylated proteins [442]. These findings highlight the potential role of metal ions as key mediators of specific cell death pathways, dependent on inter-organelle communication.

Collectively, these results demonstrate the interactions between different forms of RCD, collectively determining cellular fate under specific conditions. Understanding the mechanisms governing the balance between RCD pathways is essential for deciphering the perpetual battle between cell survival and demise.

## Conclusions and outlooks

Ferroptosis, characterized by iron-dependent lipid peroxidation, represents a distinct form of RCD that has captured the interest of researchers due to its unique mechanisms and biological significance. The discovery of ferroptosis has not only expanded our understanding of cell death pathways but has also opened new avenues for therapeutic intervention, particularly in diseases where dysregulated cell death is prominent, such as cancer and neurodegenerative disorders. Researchers are actively exploring pharmacological and genetic strategies to modulate ferroptosis for therapeutic benefit.

Despite its promise, ferroptosis research faces several challenges that hinder our ability to fully understand and manipulate this cell death pathway. Accurately delineating the complex molecular mechanisms governing ferroptosis initiation, propagation, and regulation poses a significant challenge, as does identifying reliable biomarkers specific to ferroptosis, particularly in vivo. Discriminating ferroptosis from other forms of RCD and deciphering its role in various physiological and pathological conditions also present considerable hurdles. Although lipid peroxidation is considered a central event driving ferroptosis, it is also implicated in other forms of RCD. It also remains unclear how traditional chemotherapy or radiation therapy induce apoptosis, while now also potentially triggering ferroptosis. The ferroptosis inducer and inhibitor exhibit varying activities across different disease models, suggesting that the underlying mechanisms may differ. Additionally, translating basic research findings into clinically relevant therapeutic strategies requires overcoming barriers related to drug delivery, specificity, and potential off-target effects.

In the future, ferroptosis research is poised to advance along several promising directions. Key focuses include elucidating the precise molecular mechanisms and regulatory networks governing ferroptosis across diverse cellular contexts and disease states. This involves further exploration of the interplay between iron metabolism, lipid peroxidation, antioxidant defenses, and signaling pathways implicated in ferroptotic cell death. Efforts will also continue to identify and validate novel biomarkers specific to ferroptosis for diagnostic and therapeutic purposes. Moreover, research will increasingly explore the therapeutic potential of targeting ferroptosis in various diseases, leveraging cutting-edge technologies to discover new ferroptosis modulators and enable precision medicine approaches tailored to individual patient profiles.

Specifically, several critical areas warrant attention. Firstly, understanding the causal relationship between ferroptosis and disease at the genetic level in clinical settings is challenging. Standardized animal models are necessary to enhance our understanding of ferroptosis biology, particularly regarding potential side effects and drug resistance associated with different dosing regimens.

Secondly, enhancing the bioavailability and targeting of ferroptosis inducers is crucial for clinical dissemination. Strategies, such as PROTACs and lysosomal-targeted chimaeras, hold promise in this regard [443]. Shifting the focus of targeting from complete ablation of GPX4 to other ferroptosis-regulating complexes with fewer side effects could be beneficial.

Thirdly, integrating patient data to pinpoint individuals who would benefit most from ferroptosis therapy is essential. Analyzing genomic differentiation among patients requires careful consideration of ethnographic differences and errors due to sample size.

Fourthly, promising applications of ferroptosis in different diseases, particularly cancer, are emerging. Combining different strategies, such as dietary interventions, can significantly improve treatment outcomes with fewer side effects. Future comprehensive safety assessments of combining ferroptosis with dietary interventions hold promise for cancer treatment [100, 277, 444].

Fifthly, developing cell-specific precision-targeted inducers of ferroptosis remains challenging. Strategically targeting immune cells and cancer cells for drug development, leveraging variances in PUFA-PL content, proves to be a promising approach [445].

Finally, despite a growing number of drugs inducing ferroptosis in cancer cells, distinguishing ferroptosis from other RCD remains crucial. Numerous anticancer agents have the potential to trigger a mixed type of cell death. Changes in specific proteins or biochemical indicators alone are insufficient to classify cell death as ferroptosis. Comprehensive assessments, including multidimensional analyses utilizing single-cell genomics, spatial transcriptomics, proteomics, and metabolomics, are needed for effective classification.

Overall, interdisciplinary collaboration, technological innovation, and a deeper understanding of ferroptosis biology will drive the field forward, offering new insights into disease pathogenesis and potential avenues for therapeutic intervention.

## Data Availability

No datasets were generated or analysed during the current study.
